# In vitro comparative evaluation of disinfectant-loaded nanoparticles against biofilm-forming *Vibrio* spp. isolated from gilthead seabream (*Sparus aurata*)

**DOI:** 10.1038/s41598-026-45352-0

**Published:** 2026-04-15

**Authors:** Esraa Tawfeek Ismail, Mai A. M. El-Son, Wafaa Ragab, Hazem Ramadan, Fatma A. El-Gohary, Eman Zahran

**Affiliations:** 1 Department of Aquatic Animal Medicine, Faculty of Veterinary Medicine, Mansoura University, Mansoura, 35516 Egypt; 2 Horus Research Center, Horus University – Egypt (HUE), New Damietta, 34518 Egypt; 3 Department of Bacteriology, Immunology and Mycology, Faculty of Veterinary Medicine, Mansoura University, Mansoura, Egypt; 4 Department of Hygiene and Zoonoses, Faculty of Veterinary Medicine, Mansoura University, Mansoura, 35516 Egypt

**Keywords:** Bacterial pathogens, Antibiofilm, MIC, MBC, Fish, Biotechnology, Microbiology

## Abstract

This study evaluated the in vitro efficacy of antimicrobial and antibiofilm of three disinfectant-loaded nanoparticles (AgNPs-H₂O₂, CuNPs-Virkon S, and CuNPs-TH_4_) against *Vibrio alginolyticus* and *Vibrio fluvialis,* isolated from naturally infected gilthead seabream in Egypt. Qualitative Congo red assay showed that *V. fluvialis* isolates were strong biofilm producers, whereas *V. alginolyticus* showed limited phenotypic variation. Quantitative assay results, analyzed with a Mann–Whitney U test, showed no statistically significant difference in overall biofilm biomass between *V. alginolyticus* and *V. fluvialis* isolates (U = 157.0, *p* = 0.175). Seasonal variation affected biofilm formation, peaking in summer. CuNPs-TH4 exhibited the highest antibiofilm effect, significantly reducing the biomass (*p* < 0.01). The Minimum Inhibitory Concentration (MIC) was significantly different between disinfectants (*p* < 0.001), with CuNPs-TH_4_ having the highest MIC values, followed by AgNPs-H₂O₂ and CuNPs-Virkon S. Minimum bactericidal concentration (MBC) values showed that AgNPs-H₂O₂ had the most consistent bactericidal activity in 85.4% of isolates tested (defined by MBC/MIC ≤ 4). A significant association was observed between MIC values and *Vibrio* species (p = 0.002). Molecular detection of antimicrobial resistance (AMR) genes revealed a high prevalence of *sul*, *cat*, and *mphA*, with *V. alginolyticus* exhibiting a broader resistance profile than *V. fluvialis*. These findings highlight the in vitro potential of nanotechnology-enhanced disinfectants, within the limitations of nanoparticle-only testing, as exploratory alternatives for controlling biofilm-associated ***Vibrio*** infections in aquaculture.

**Supplementary Information:**

The online version contains supplementary material available at 10.1038/s41598-026-45352-0.

## Introduction

Aquaculture has become one of the most effective solutions to address global food scarcity, contributing significantly to economic growth and employment, particularly in Egypt^1,2^. However, the sustainability of aquaculture production is increasingly threatened by disease outbreaks caused by microbial pathogens threaten the sustainability of aquaculture production^3^.

*Vibriosis* is a major bacterial disease that affects marine aquaculture worldwide, leading to high mortality rates and substantial economic loss^4^. *Vibrio* spp. are naturally distributed in the coastal and estuarine environments. Biofilm-forming *Vibrio* strains are of particular concern because biofilm formation significantly reduces the efficacy of antimicrobial agents and promotes the emergence of antibiotic resistance^5^. A biofilm is a structured community of bacterial cells encased in a self-produced extracellular polysaccharide (EPS) matrix that protects bacteria from environmental stress and antimicrobial penetration^6^. Therefore, the development of novel antimicrobial strategies capable of eradicating biofilms has become a major priority for aquatic disease control.

Among *Vibrio* species, *V. alginolyticus* is one of the most prevalent marine pathogens, infecting several cultured fish species, including mullet *(Mugilidae spp.),* European seabass *(Dicentrarchus labrax),* gilthead seabream *(Sparus aurata)*^7^, large yellow croaker *(Larimichthys crocea)*^8^, and Nile tilapia *(Oreochromis niloticus)*^9^. *V. fluvialis* is an emerging human pathogen associated with foodborne infections and has been isolated from marine organisms such as mussels and oysters^10,11^. It has also been recovered from cultured fish, including thin-lip gray mullet (*Liza ramada*), gilthead seabream, and European seabass^12,13^.

The excessive use of antibiotics in aquaculture to control *Vibrio* infections has led to the emergence of multidrug-resistant strains and environmental contamination. The misuse of antimicrobials not only disrupts aquatic microbial communities but also poses serious zoonotic threats^14,15^. In *Vibrio* species, antimicrobial resistance is commonly mediated by transferable gene-encoding mechanisms, such as enzymatic inactivation, efflux pumps, and target-site modifications^16^.

Chemical disinfectants are an important category in the biosecurity program, including hatcheries, larval rearing units, grow-out ponds, and net-cages^17^. Hydrogen peroxide is one of the widely used egg disinfectants and water treatment owing to its powerful oxidizing capacity and ecofriendly degradable byproduct^18^.Virkon S, a peroxygen-based disinfectant with broad-spectrum activity, and TH4, a quaternary ammonium compounds, are mostly used for equipment and facility sanitization^19^. Regardless of their widespread use, their efficacy against biofilm-forming *Vibrio* species is still variable, especially in resistant strains^20^. Therefore, integrating these disinfectants with nanoparticle-based delivery systems may improve their antibiofilm activity and effectiveness under laboratory conditions.

Various antimicrobial resistance genes (ARGs) have been frequently detected in *Vibrio* spp. in aquaculture and seafood environments, including resistance to sulfonamides (*sul1, sul2*)^21,22^, aminoglycosides (*armA*, *aac(3)-IIa*, and *strA-strB*)^23^, phenicols (*cat* and *floR)*^24^, and tetracyclines *(tet)*^25^. Macrolide resistance mediated by (*mphA*) has been described in clinical *V. fluvialis* isolates, demonstrating its potential for horizontal spread^26^. Such studies emphasize that *V. alginolyticus* and *V. fluvialis* are opportunistic pathogenic species with a high prevalence of antimicrobial resistance, underscoring the importance of monitoring these pathogens in marine aquaculture and the environment. Furthermore, the use of alternative antimicrobial agents that target antibiotic resistance by preventing biofilm formation has yielded promising results^27^.

Recently, nanotechnology has emerged as a rapidly developing interdisciplinary field that offers innovative approaches for synthesizing nanoparticles with potent antimicrobial and antibiofilm activities. Owing to their unique physicochemical properties, enhanced cell membrane permeability, and multitarget modes of action, nanoparticles have shown enhanced activity in some contexts than conventional antibiotics against drug-resistant pathogens^28–30^.

The antimicrobial efficacy of several metallic nanoparticles, including silver, copper, zinc, and titanium, has been widely reported^31^. *V. alginolyticus,* and other *Vibrio* spp*.,* such as *V. parahaemolyticus, V. harveyi, V. vulnificus,* and *V. cholerae,* were found to be susceptible to the effects of nanomaterials, including silver, copper, zinc oxide, titanium dioxide, and silver-doped zeolites^32–36^.

Among metallic nanoparticles, copper nanoparticles (CuNPs) are widely recognized for their antimicrobial ability against a variety of pathogens and as potential antibiofilm agents^37–39^, Silver nanoparticles (AgNPs) are broad-spectrum agents effective against both Gram-negative and Gram-positive bacteria, fungi, and viruses^40–43^.

*Vibriosis,* primarily caused by *V. alginolyticus* and *V. fluvialis*, continues to threaten gilthead sea bream (*Sparus aurata*) aquaculture, resulting in considerable economic losses. Our previous research revealed the seasonal prevalence, genetic diversity, and antimicrobial resistance profiles of these pathogens in coastal farms in Damietta, Egypt, where multidrug resistance indices (MAR) ranged from 0.3 to 0.7^44^. Given the escalating issue of antibiotic resistance, alternative strategies, such as nanotechnology-based disinfectants, are urgently needed to control diseases in aquaculture. This study builds upon our previous synthesis work^45^; therefore, the innovative aspect of the present work lies in its application to *Vibrio* isolates from marine fish with specific biofilm phenotypes, thereby providing host-specific data essential for future field validation, rather than in nanomaterial development. The present study aimed to evaluate the in vitro efficacy of selected disinfectant-loaded hydrogen peroxide-loaded silver nanoparticles (AgNPs-H₂O₂), Virkon S-loaded copper nanoparticles (CuNPs-Virkon S), and TH4-loaded copper nanoparticles (CuNPs-TH4) against multidrug-resistant, biofilm-producing *Vibrio* spp. isolated from seabream fish, providing insight into potential solutions for managing vibriosis in aquaculture systems. Unlike previous studies focusing on the antimicrobial activity of free disinfectants or nanoparticles alone, the present study introduces a disinfectant-loaded nanoparticle system designed to enhance antibiofilm efficacy against fish pathogenic bacteria. The novelty of this approach lies in improving disinfectant stability and localized activity within biofilm matrices, thereby reducing the effective concentration required for biofilm inhibition.

## Materials and methods

### Bacterial isolates

A total of 205 V*. alginolyticus* and 62 V*. fluvialis* isolates were previously recovered from 115 gilthead seabream from coastal farms in Damietta, Egypt, on a seasonal basis over a 10-month period (September 2022 to July 2023) as described in our earlier stud^44^. Briefly, bacterial isolation was performed on TCBS agar, followed by biochemical identification using the DL D2mini Microbial ID and AST system. *V. alginolyticus* isolates were molecularly characterized, but *V. fluvialis* identification was based only on consistent biochemical profiles using DL D2mini Microbial ID and AST system **(**ZHUHAI DL BIOTECH Co., Ltd), according to the manufacturer’s instructions. Additionally, clustering patterns were used to ensure representative diversity, where 53 V*. alginolyticus* and *V. fluvialis* selected isolates were genotyped by ERIC-PCR, as described in our earlier study^44^. For the present study, a representative subset of isolates was selected purposively, not randomly, to ensure representation based on (i) ERIC-PCR clustering patterns for both isolates (Supplementary Figure S1), (ii) consistent biochemical profiles (Supplementary Table S1), (iii) antimicrobial resistance patterns, and (iv) biofilm-forming phenotype. This strategy was adopted to ensure diversity while maintaining feasibility for labor-intensive in vitro assays, including MIC, MBC, time–kill kinetics, and molecular detection of antimicrobial resistance genes, providing a robust preliminary assessment for future large-scale studies.

### Assessment of biofilm formation for isolated *Vibrio spp.*

In this section, A Congo red agar (CRA) assay was used solely as a qualitative phenotypic screening tool for biofilm production. In contrast, quantitative biofilm classification, statistical analysis, and intergroup comparisons were based solely on the microtiter plate assay (MTPA). Discrepancies between CRA pigmentation patterns and MTPA-derived biofilm strength were therefore anticipated, as the two methods differ substantially in sensitivity, specificity, and underlying detection principles. The biofilm-forming ability of 33 *Vibrio* isolates (11 V*. alginolyticus* and 22 V*. fluvialis*) was evaluated qualitatively using the Congo red agar (CRA) method, with brief modifications. The CRA was prepared with 37 g/L brain heart infusion broth (BHI, Biokar Diagnostics), 50 g/L sucrose, 10 g/L agar, and 0.8 g/L Congo red (MP Biomedicals, LCC, France), as described by Milanov, et al.^46^, to phenotype biofilm-producing isolates. *Vibrio* strains were initially cultured overnight in BHI at 28 °C. Subsequently, the bacterial cultures were streaked onto CRA plates in a zigzag pattern and incubated at 28 °C for 24 h. Following incubation, the plates were visually inspected for black pigment formation, a sign of biofilm production. Method validity was confirmed using reference strains (*Staphylococcus aureus* ATCC 35,984, positive; *S. epidermidis* ATCC 12,228, negative).

The biofilm-forming ability of these isolates was assessed using a microtiter plate assay (MTPA) in 96-well plates, following a previously described protocol O’Toole^47^ with minor modifications. Briefly, tested isolates were grown overnight, adjusted to the desired cell density, and inoculated into three independent wells (triplicate manner) of sterile 96-well microtiter plates. A positive control (defined biofilm-forming strain) and a negative control (sterile broth without bacterial isolates) were inoculated in each plate. After incubation, planktonic cells were discarded, and wells were washed three times with sterile phosphate-buffered saline (PBS). Adherent biofilms were fixed and stained with 0.1% (w/v) crystal violet solution for 15 min. Excess stain was removed, and wells were rinsed thoroughly with distilled water. The bound crystal violet was solubilized using 95% ethanol, and absorbance was measured using a microplate reader. Biofilms were then quantified by measuring the optical density (OD) at 620 nm, which corresponds to the available microplate reader filter setting used in this study. Previous studies have demonstrated that crystal violet–based biofilm quantification can be reliably performed over a wavelength range of 550–620 nm, depending on instrument configuration, without affecting relative biofilm classification^48,49^. Biofilm formation capacity was interpreted according to the criteria described by Stepanović, et al.^49^, where OD > 4 × ODc indicates strong biofilm formation, 2 × ODc < OD ≤ 4 × ODc indicates moderate biofilm formation, ODc < OD ≤ 2 × ODc indicates weak biofilm formation, and OD < ODc denotes non-biofilm producers. The cut-off OD (ODc) was defined as the mean absorbance of the negative control (medium without cells).

### Preparation and characterization of disinfectant nanocomposites

Three disinfectant nanocomposites (hydrogen peroxide-loaded silver nanoparticles (AgNPs-H_2_O_2_), Virkon S-loaded copper nanoparticles (CuNPs-Virkon S), and TH_4_-loaded copper nanoparticles (CuNPs-TH_4_) were assessed for their efficacy against biofilm-forming *Vibrio* isolates. **The disinfectant-loaded nanoparticles used in this study were synthesized and characterized as previously described by Elsayed, et al.**^45^ (Supplementary Figures S2-4). In brief, silver and copper nanoparticles (AgNPs and CuNPs) were synthesized using AgNO_**3**_** and CuSO**_**4**_**, respectively, with a focus on employing benign natural polyphenols as co-stabilizers instead of harmful artificial stabilizers. The morphology and size of the synthesized nanocomposites were characterized using TEM and zeta potential analysis. These nanoparticles were then incorporated into five commercial disinfectants (DC&R®, VirkonS®, TH4 +  + , Tek-Trol, and peracetic acid) commonly used in fish farms.** No modifications were made to the original nanoparticle formulation.

### Determinations of minimum inhibitory concentration (MIC) and minimum bactericidal concentration (MBC)

Due to the labor-intensive nature of Minimum Inhibitory Concentration (MIC) and Minimum Bactericidal Concentration (MBC), and time-kill assays, a reduced number of representative isolates (n = 10) was selected to reflect species diversity, biofilm phenotype, and resistance profiles and determined following the guidelines outlined by the Clinical and Laboratory Standards Institute (CLSI)^50^. The MIC was assessed using the broth microdilution method following standard protocols. Briefly, bacterial colonies were adjusted to an optical density (OD_625_) between 0.08 and 0.12, corresponding to approximately 10^^6^ colony-forming units (CFU) per milliliter, using Tryptic Soy Broth (TSB). Tryptic soy broth (TSB, Oxoid- UK) can be used for antimicrobial and MIC assays for different bacterial pathogens, including *Vibrio* species, as documented in previous studies. Moreover, we used TSB for *Vibrio* species owing to its richness in nutrients and ability to foster the bacterial broth during susceptibility testing^51^. Disinfectant mixtures were prepared at various concentrations using two-fold serial dilutions in 96-well microtiter plates. Each well was seeded with 50 µL of TSB and 50 µL of bacterial suspension. Subsequently, 100 µL of the prepared disinfectant solution was added to the first well, and subsequent dilutions were transferred to consecutive wells, resulting in a range of concentrations (50, 25, 12.5, 6.25, 3.125, 1.563, 0.781, 0.39, 0.195, and 0.098 µg/ml). The plates were then incubated at 28 °C for 24 h. The MIC was determined as the lowest concentration of disinfectant-loaded nanoparticles at which no visible bacterial growth occurred. Control wells without bacterial growth (Well 1) and with bacterial growth (Well 12) were used for comparison, for data verification, the MIC of each isolate was determined in triplicate. The MIC and MBC were determined using the broth microdilution method following the guidelines of the Clinical and Laboratory Standards Institute (CLSI)^50^.

Following MIC determination, the MBC was determined by subculturing 50 µL from each well that exhibited no visible turbidity on Tryptic Soy Agar (TSB, Oxoid, UK) plates. The plates were then incubated at 28 °C for 24 h and the presence or absence of bacterial growth was assessed. MBC was defined as the lowest concentration of disinfectant-loaded nanoparticles that completely inhibited bacterial growth on TSA plates. The MBC/MIC ratio, measured for the tested NPs products to assess their activity mode, revealed bactericidal action at scores of 1, 2, and 4, and bacteriostatic action at scores > 4^52^.

### Time killing assay (time- killing curve)

The dynamic bactericidal activity of the nanoparticle formulations was evaluated using a time–kill assay as previously described by El-Gohary, et al.^53^, with minor modifications. A total of 10 multidrug-resistant biofilm-producing *Vibrio* isolates (five *V. alginolyticus* and five *V. fluvialis*) were tested against three products: H₂O₂-loaded AgNPs, TH4-loaded CuNPs, and Virkon S-loaded CuNPs. For each isolate and product, the assay was performed at concentrations equivalent to 0.25 × MIC, 0.5 × MIC, and 1 × MIC, along with two controls: a growth control (untreated culture) and a negative control (medium without bacterial inoculation). Briefly, following MIC determination, bacterial suspensions were prepared in TSB at a final concentration of ~ 5 × 10^^5^ CFU/mL, Aliquots of 1 mL of cultures were taken at time intervals of (0, 15, 30, 60, and 120 min)^54^. At each interval, viable bacterial counts were determined by plating on TSA (TSA, Oxoid, UK) using the spread plate method, and the colony-forming units (CFU/mL) were enumerated. Viable bacterial counts were converted to log₁₀ CFU/mL prior to plotting. When no colonies were detected, values were considered below the detection limit and treated accordingly for graphical representation. The results were plotted to compare the bactericidal kinetics of each nanoparticle formulation against the positive and negative controls^55^.

### Molecular detection of antibiotic resistance genes (ARGs) in isolated *vibrio* species

Polymerase chain reaction (PCR) analysis was performed to screen for different antimicrobial resistance genes in *Vibrio* isolates (N = 10), using targeted specific primer pairs. Genomic DNA was extracted from overnight bacterial cultures using the boiling lysis method according to Ahmed and Dablool^56^. PCR amplification was performed using a Bio-Rad T100™ Thermal Cycler ((Bio-Rad Laboratories, Hercules, CA, USA). Each 25 µL PCR reaction consisted of 12.5 µL of 2X PCR Master Mix, 1 µL of each forward and reverse primer (10 µM), 2 µL of genomic DNA template, and 8.5 µL of nuclease-free water. The optimized cycling conditions were as follows: initial denaturation at 95 °C for 5 min, 35 cycles of (95°) C for 1 min, annealing temperatures as specified for each primer for 1 min (Table 1), and extension at 72 °C for 1 min. The final extension step was performed at 72 °C for 7 min. Five microliters of PCR products were electrophoresed on a 1.5% agarose gel in 1 × TAE buffer at 100 V for 45 min, then visualized under UV light using a UV transilluminator (Spectroline, USA) and sized against a 100 bp DNA ladder. The selected target genes were as follows: sulfonamide resistance genes (sul1), tetracycline resistance genes (tetA), florfenicol resistance genes (floR), TEM-type β-lactamase (blaTEM), chloramphenicol acetyltransferase gene (cat), OXA-type β-lactamase (blaOXA), macrolide phosphotransferase gene (mphA), and CTX-M β-lactamase (blaCTX-M). The detection of multiple antimicrobial resistance genes in several isolates indicates a potential multidrug resistance phenotype. Multidrug resistance (MDR) is defined as resistance to at least one antimicrobial agent in three or more antimicrobial classes^57^. **Mapping of detected resistance genes to corresponding antibiotic classes is presented in **Table 2**.**

**Table 1:** The primer sets used for AMR genes detection.

Gene	Forward	Reverse	References
*sul1*	TCACCGAGGACTCCTTCTTC	CAGTCCGCCTCAGCAATATC	Chen et al., 2004^100^
*tetA*	GCGCCTTTCCTTTGGGTTCT	CCACCCGTTCCACGTTGTTA	Chen et al., 2004^100^
*floR*	CTGAGGGTGTCGTCATCTAC	GCTCCGACAATGCTGACTAT	Chen et al., 2004^100^
*blaTEM*	ATAAAATTCTTGAAGACGAAA	GACAGTTACCAATGCTTAATC	Ahmed et al., 2006^101^
*cat1*	CTTGTCGCCTTGCGTATAAT	ATCCCAATGGCATCGTAAAG	Chen et al., 2004^100^
*blaOXA*	TATCTACAGCAGCGCCAGTG	CGCATCAAATGCCATAAGTG	Ouellette et al., 1987^102^
*mphA*	GTGAGGAGGAGCTTCGCGAG	TGCCGCAGGACTCGGAGGTC	Phuc Nguyen et al., 2009^103^
*blaCTX*	CGCTTTGCGATGTGCAG	ACCGCGATATCGTTGGT	Ahmed et al., 2006^101^

**Table 2:** Mapping of detected resistance genes to corresponding antibiotic classes.

Gene	Antibiotic class	Corresponding antibiotic in AST
Sul	sulfonamides	Sulfamethoxazole
Tet	tetracycline	Tetracycline
FloR	phenicol	Florfenicol
Cat	phenicol	Chloramphenicol
TEM / OXA / CTX	β-lactam	Amoxicillin
mphA	macrolide	Erythromycin

### Statistical analysis

Relationships between categorical variables were analyzed using cross-tabulation and chi-square tests in SPSS 23, with significance at p < 0.05. Descriptive statistics, including frequencies and percentages, summarized data. Given the small sample size and sparse tables, categorical associations were explored descriptively using chi-square tests, with results interpreted cautiously. A p-value < 0.05 was considered statistically significant. The prevalence of bacterial isolates (including biofilm producers) was calculated as follows: Prevalence (%) = (number of selected isolates/total bacterial isolates) × 100. Seasonal variations in prevalence were assessed using the chi-square test, with P < 0.05 indicating significance.

## Result

### Detection of biofilm-forming strains using modified Congo red agar media (CRAM)

**Fig. 1:**
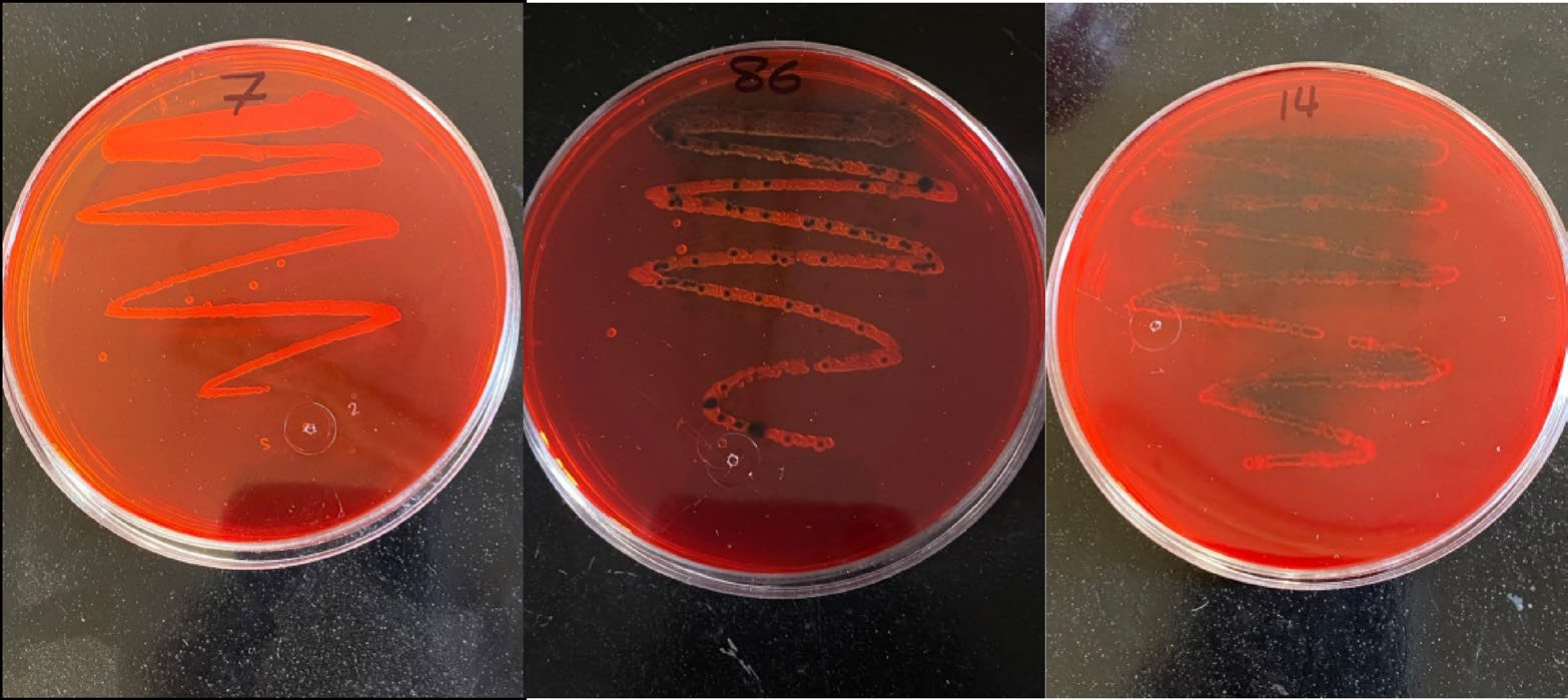
Biofilm formation ability of *V. alginolyticus* and *V. fluvialis* on Congo agar media.

**Fig. 2:**
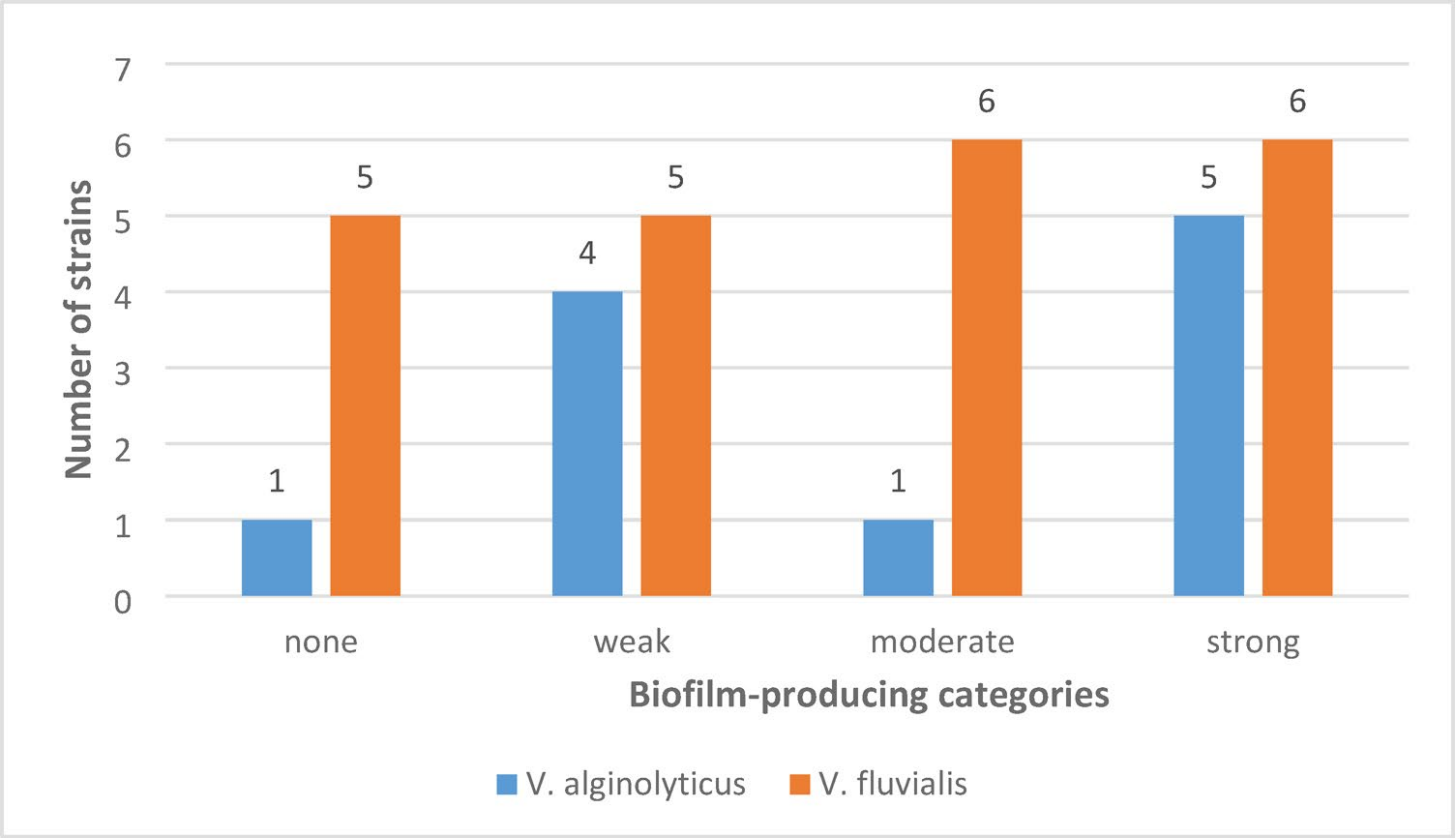
Biofilm phenotypes distribution of *Vibrio* spp. strains isolated from infected seabream.

CRA pigmentation patterns were interpreted as qualitative indicators of biofilm phenotype and were not used to define quantitative biofilm strength, which was assessed based on MTPA. The modified Congo Red Agar (CRA) medium distinguishes biofilm producers from non-producers based on the colony appearance. As illustrated in Fig. 1, colonies exhibiting dark to light black pigmentation were indicative of strong biofilm formation, whereas bright red colonies denoted non-biofilm-producing strains. Strains were categorized as non-biofilm, weak, medium, and strong biofilm producers. Most isolates of *V. alginolyticus* were strong biofilm producers (5/11), followed by weak producers (4/11), while only one isolate each was classified as a non-producer (1/11) or a moderate producer (1/11). In contrast, *V. fluvialis* showed higher numbers across all categories, with the greatest frequency observed in the moderate and strong biofilm classes (6 isolates each), followed by weak producers (5 isolates) and non-producers (5 isolates), indicating that strong biofilm producers were observed mainly among *V. fluvialis* isolates, whereas *V. alginolyticus* showed limited phenotypic variation **(**Fig. 2**)**.

### Biofilm formation ability of *Vibrio* isolates in microtiter plates

**Fig. 3:**
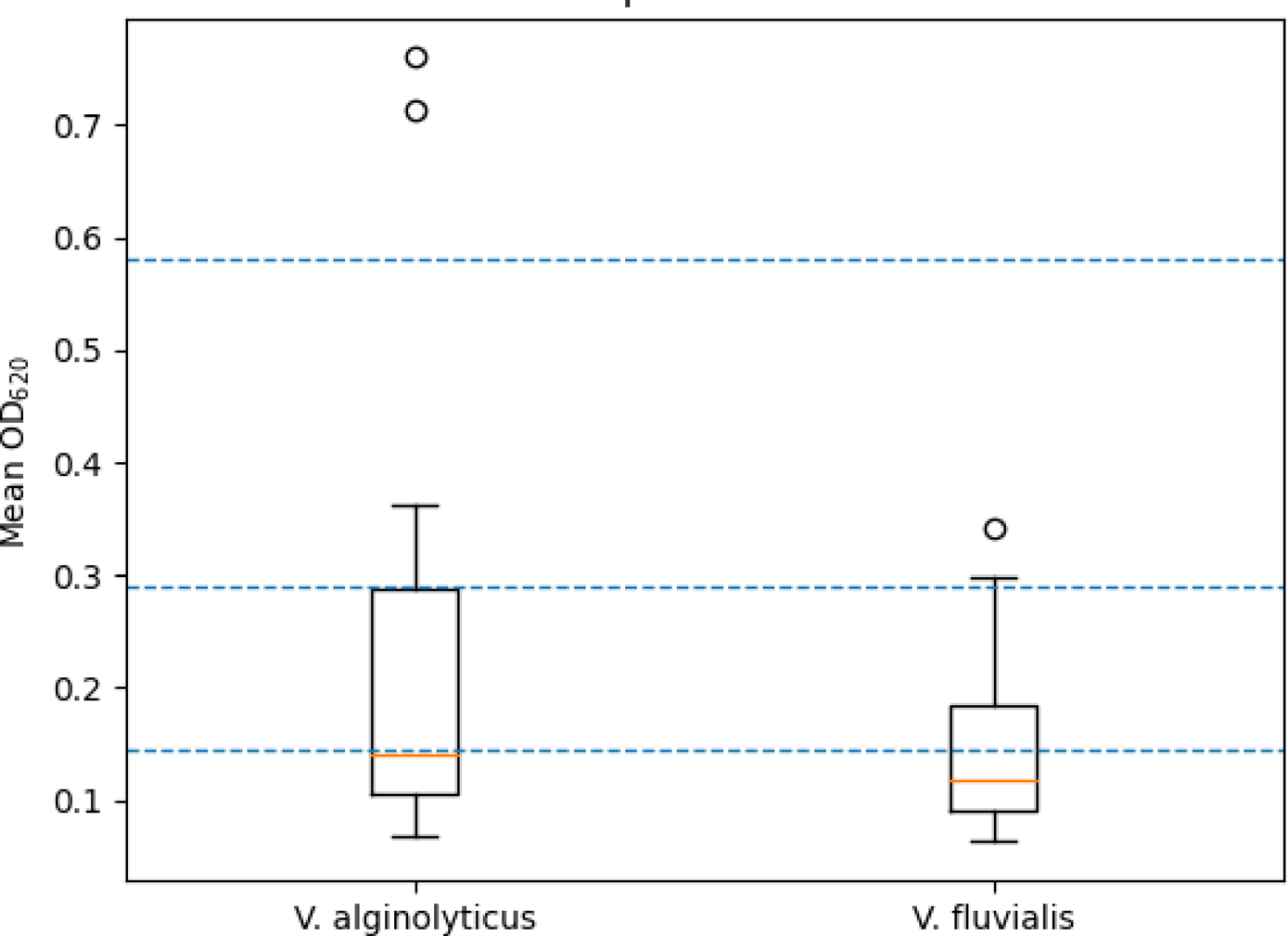
Box-and-whisker plot showing the distribution of mean OD₆₂₀ values per isolate for *Vibrio alginolyticus* (n = 11) and *Vibrio fluvialis* (n = 22). Horizontal dashed lines represent the biofilm classification thresholds defined by Stepanović et al. (2007): ODc (0.145), 2 × ODc, and 4 × ODc. Boxes represent the interquartile range, horizontal lines indicate the median, whiskers show minimum–maximum values, and open circles indicate outlier isolates exhibiting elevated biofilm biomass.

Because the Congo red agar (CRA) assay provides only a qualitative assessment of biofilm phenotype, these results were considered preliminary indicators and were not used alone to rank biofilm strength; quantitative classification was based exclusively on microtiter plate assay results. Biofilm biomass was quantified using the microtiter plate assay and interpreted according to the Stepanović, et al.^49^ classification scheme. Based on OD₆₂₀ cut-off values (ODc = 0.145), the majority of isolates from both species were classified as weak biofilm producers, whereas only a limited number exceeded the 2 × ODc and 4 × ODc thresholds and were categorized as moderate or strong biofilm producers. Distribution-based comparison using a Mann–Whitney U test revealed no statistically significant difference in overall biofilm biomass between *V. alginolyticus* (0.264 ± 0.236) and *V. fluvialis* (0.141 ± 0.073) isolates (U = 157.0, p = 0.175). A small number of isolates displayed markedly elevated OD₆₂₀ values and appeared as outliers in the distribution plots, reflecting strong biofilm-forming phenotypes (Fig. 3).

### Seasonal prevalence of biofilm-forming *Vibrio* strains isolated from naturally diseased seabream

For *V. alginolyticus*, biofilm-positive isolates were most commonly detected during summer (22/71, 30.9%), followed by winter (9/51, 17.6%) and autumn (4/33, 12.1%), whereas no biofilm-forming isolates were detected during spring. In contrast, *V. fluvialis* displayed the highest proportion of biofilm-positive isolates during summer (9/13, 69.2%), with progressively lower proportions in autumn (10/21, 47.6%), winter (9/26, 34.6%), and spring (5/27, 18.5%). Overall, seasonal differences in the frequency of biofilm-positive isolates were statistically significant (χ^2^ test, *p* = 0.0075). Some seasonal categories contained a limited number of isolates; therefore, results should be interpreted carefully and are presented primarily to indicate temporal trends rather than definitive population-level associations. Detailed prevalence data for the total and biofilm-forming isolates across the different seasons are presented in Table 3.

**Table 3:** The seasonal biofilm formation rate of *Vibrio spp.*

Season	*V. alginolyticus*		*V. fluvialis*	
	Prevalence % (n/N)*	Biofilm forming rate % (n/N)	Prevalence % (n/N)	Biofilm forming rate % (n/N)
Autumn	39.75% (33/83)	4/33 (12.1%)	25.30% (21/83)	10/21 (47.6%)
Winter	49.51% (51/103)	9/51 (17.6%)	25.24% (26/103)	9/26 (34.6%)
Spring	40.65% (50/123)	0	21.95% (27/123)	5/27 (18.5%)
Summer	57.72% (71/123)	22/71 (30.9%)	10.56% (13/123)	9/13 (69.2%)

*n = number of isolates in the specified category; *N* = total number of isolates recovered in the corresponding season. Biofilm positivity was determined based on microtiter plate assay results and reflects the presence or absence of biofilm formation rather than quantitative biomass intensity.

**Seasonal variation in the proportion of biofilm-forming isolates was statistically significant (χ^2^ test, p = 0.0075).

### MIC and MBC of the examined disinfectant-loaded nanoparticles against isolated *Vibrio* strains.

Comparative antimicrobial activity profiles of nanomaterial–disinfectant composites against *Vibrio* isolates are presented in Table 4. The MIC values of silver nanoparticles combined with hydrogen peroxide (AgNPs–H₂O₂) ranged from 6.25 to 25 µg/mL against the tested *Vibrio* isolates. Copper nanoparticles combined with Virkon S (CuNPs–Virkon S) showed lower MIC values, ranging from 1.563 to 12.5 µg/mL, indicating stronger inhibitory activity. In contrast, MIC values for copper nanoparticles combined with TH4 (CuNPs–TH4) ranged between 6.25 and 25 µg/mL. The MBC values for all nanomaterial–disinfectant formulations ranged from 12.5 to 50 µg/mL across the tested isolates. The bactericidal efficacy was further evaluated using the MBC/MIC ratio. Most isolates exhibited MBC/MIC ratios ≤ 4, indicating predominantly bactericidal activity, although higher ratios (≥ 8) were observed in a limited number of cases, particularly for AgNPs–H₂O₂ and CuNPs–Virkon S, suggesting reduced bactericidal efficiency in some strains. Crosstab analysis using Pearson’s chi-square test demonstrated distinct antimicrobial response patterns among the formulations. No significant associations were observed between bacterial type and disinfectant product (χ^2^ = 0.048, p = 1.000), tested concentration (χ^2^ = 0.000, p = 1.000), or MBC values (χ^2^ = 8.928, p = 0.178). However, a statistically significant association was detected between bacterial type and MIC values (χ^2^ = 20.569, p = 0.002), indicating strain-dependent variation in susceptibility. Significant differences in MIC distributions were observed among the nanomaterial–disinfectant composites (χ^2^ = 18.852, df = 2, p < 0.001). CuNPs–TH4 exhibited the highest MIC values, with 80.6% of isolates falling within higher MIC categories, followed by AgNPs–H₂O₂ (75.9%), while CuNPs–Virkon S showed the lowest MIC values (64.0%), reflecting superior inhibitory efficacy. MBC values also differed significantly among formulations (χ^2^ = 6.64, df = 2, p = 0.036). AgNPs–H₂O₂ demonstrated the strongest bactericidal activity, with 85.4% of isolates exhibiting lower MBC values, followed by CuNPs–TH4 (83.0%). CuNPs–Virkon S showed comparatively lower bactericidal efficacy (77.1%). These findings indicate significant formulation-dependent differences in both inhibitory and bactericidal activities against *Vibrio* isolates. Nevertheless, due to the limited number of tested isolates, these results should be interpreted as exploratory and warrant confirmation using larger datasets.

**Table 4:** Comparative antimicrobial activity profiles of nanomaterial–disinfectant composites against Vibrio isolates.

Parameter	AgNPs–H₂O₂	CuNPs–Virkon S	CuNPs–TH₄	Statistical significance
MIC range (µg/mL)	6.25–25	1.563–12.5	6.25–25	
Median MIC (µg/mL)	12.5	12.5	12.5	
Isolates with high MIC (≥ 12.5 µg/mL)	80% (8/10)	60% (6/10)	90% (9/10)	χ^2^ = 18.85, p < 0.001*
Low MIC (≤ 6.25 µg/mL)	20% (2/10)	40% (4/10)	10% (1/10)	
MBC range (µg/mL)	25–50	12.5–50	12.5–50	
Bactericidal activity (MBC/MIC ≤ 4)	90% (9/10)	80% (8/10)	90% (9/10)	χ^2^ = 6.64, p = 0.036*
Species-specific susceptibility (MIC)	No significant association	No significant association	Significant variation among species	χ^2^ = 20.57, p = 0.002*

* Statistically significant at p < 0.05 (Pearson’s chi-square test).

Bactericidal activity was defined as an MBC/MIC ratio ≤ 4. High MIC was defined as ≥ 12.5 µg/mL. Values represent percentages of 10 tested isolates per formulation.

### Time killing assay (time- killing curve)

**Fig. 4:**
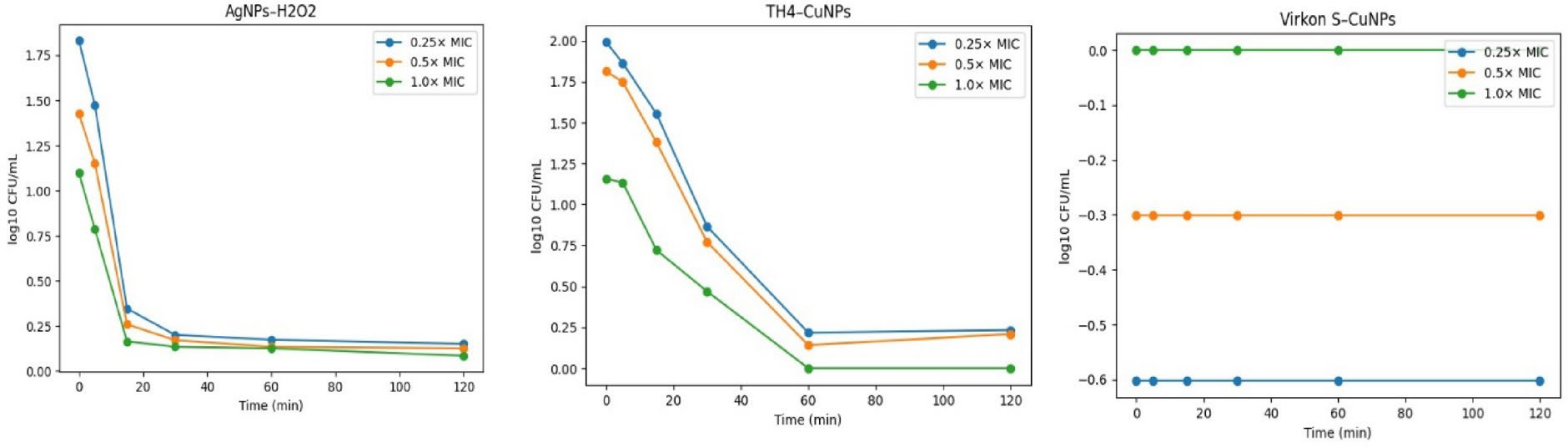
Time–kill kinetics of disinfectant-loaded nanoparticles against biofilm-producing *Vibrio* isolates. Viable counts are expressed as log₁₀ CFU/mL and plotted over time (0–120 min) at concentrations of 0.25 × , 0.5 × , and 1 × MIC. Curves represent mean values across tested isolates (n = 10). When no colonies were detected, values were considered below the detection limit and plotted accordingly.

The time-dependent bactericidal activities of the three nanoparticle formulations (H₂O₂-loaded AgNPs, TH_4_-loaded CuNPs, and Virkon S-loaded CuNPs) were assessed against 10 multidrug-resistant biofilm-producing *Vibrio* isolates (five *V. alginolyticus* and five *V. fluvialis*), as illustrated in Fig. 4. Bacterial killing kinetics were monitored by quantifying viable counts, expressed as log₁₀ CFU/mL, at 0, 15, 30, 60, and 120 min. Time–kill analysis revealed clear time- and concentration-dependent bactericidal activity of the tested disinfectant-loaded nanoparticles. AgNPs–H₂O₂ exhibited the most rapid killing kinetics, achieving near-complete bacterial inhibition within 60 min at all tested concentrations. TH4–CuNPs showed a slower but concentration-dependent bactericidal effect, with complete inhibition observed at 1 × MIC by 60 min. In contrast, Virkon S–CuNPs demonstrated limited killing activity, with minimal reductions in bacterial viability over the experimental period.

### Molecular characterization of AMR genes in tested *Vibrio* isolates

Polymerase chain reaction-based molecular characterization of ten *Vibrio* isolates revealed diverse antimicrobial resistance genes with interspecies variation (Table 5) (Supplementary Figure S5–7)*.* The sulfonamide resistance gene (*sul*) was the most prevalent, detected in 4/5 (80%) of *V. alginolyticus,* but only in 2/5 (40%) of *V. fluvialis* isolates. The *cat* gene, which is associated with chloramphenicol resistance, was similarly widespread and was present in all *V. alginolyticus* isolates 5/5 (100%) and 3/5 V*. fluvialis* isolates (60%). The macrolide resistance gene *mphA* appeared only in *V. alginolyticus* 2/5 (40%). None of the isolates tested positive for *flor*, *tet*, *OXA*, *CTX*, or *TEM*. Table 6 showed the phenotypic resistance patterns observed in the AST results from our earlier study compared with the presence of selected antibiotic resistance genes (ARGs). A clear difference was observed between the two species. *V. alginolyticus* exhibited a broader resistance gene repertoire, with most isolates harboring three AMR genes (*sul*, *cat*, and *mphA*), representing a multidrug-resistant (MDR) profile. In contrast, *V. fluvialis* isolates generally carried fewer genes, most commonly *sul* and *cat.* Additionally, some isolates resistant to β-lactams or macrolides also carried resistance determinants such as sul, cat, and mphA, suggesting a link between the detected resistance genes and the noted resistance. For example, isolates resistant to erythromycin (VA1 and VA4) were found to carry the mphA gene, which encodes a macrolide phosphotransferase and may contribute to macrolide resistance. In the same line, chloramphenicol resistance, multiple isolates harbor the cat gene, a determinant commonly associated with phenicol resistance. However, very few isolates didn’t exhibit targeted resistance genes screened in this study. Our data might suggest that other pathways or resistance mutations might be involved and were not included in our screening, indicating a partial consent between phenotypic resistance and genetic markers in the current study.

**Table 5:** Antimicrobial resistance genes detected in tested *Vibrio* isolates.

*Vibrio isolates*	*sul*	*tet*	*floR*	*TEM*	*cat*	*OXA*	*mphA*	*CTX*
VA1	** + **	**-**	**-**	**-**	** + **	**-**	** + **	**-**
*VA2*	** + **	**-**	**-**	**-**	** + **	**-**	**-**	**-**
*VA3*	**-**	**-**	**-**	**-**	** + **	**-**	**-**	**-**
*VA4*	** + **	**-**	**-**	**-**	** + **	**-**	** + **	**-**
*VA5*	** + **	**-**	**-**	**-**	** + **	**-**	**-**	**-**
*VF1*	** + **	**-**	**-**	**-**	** + **	**-**	**-**	**-**
*VF2*	** + **	**-**	**-**	**-**	**-**	**-**	**-**	**-**
*VF3*	**-**	**-**	**-**	**-**	**-**	**-**	**-**	**-**
*VF4*	**-**	**-**	**-**	**-**	** + **	**-**	**-**	**-**
*VF5*	**-**	**-**	**-**	**-**	** + **	**-**	**-**	**-**

***V. alginolyticus*** (VA1:VA5), ***V. fluvialis*** (VF1:VF5).

Positive (** +**) indicates presence of the AMR gene (positive PCR amplification); negative (**-)** indicates absence of the AMR gene (no PCR amplification detected).”

**Table 6:** Phenotypic and genotypic antimicrobial resistance profiles of *Vibrio* isolates.

*Vibrio* isolates	Antibiotics to which bacterial isolates showed resistance to	MDR pattern	MAR	ARGs detected
*V. alginolyticus isolates*				
S3 **(VA1)**	AX, E	No	0.3	sul, cat, mphA
S4 **(VA2)**, S43 **(VA5)**	AX, E, FFC, C	Yes	0.7	sul, cat
S10 **(VA3)**,	AX, E, FFC, C	Yes		cat
S17 **(VA4)**,	AX, E, FFC, C	Yes		sul, cat, mphA
*V. fluvialis* isolates				
S8 **(VF1)**,	AX, E	No	0.3	sul, cat
S42 **(VF2)**	AX, E	No	0.3	sul
S33 **(VF4)**, S47 **(VF5)**	AX, E, C	Yes	0.5	cat
S32 **(VF3)**	AX, E, FFC, C	Yes	0.7	none

*S denotes the isolates’ number with their AST based on our previous study (Ismail et al. 2024).

*V. alginolyticus (VA1:VA5), ***V. fluvialis*** (VF1:VF5), corresponding to the (S) isolates showing the AST.

## Discussion

Aquaculture is vital to global food security but continues to face major challenges from infectious diseases caused by multidrug-resistant *Vibrio* species^58^, These pathogens are particularly problematic due to their dual threat: their ability to form biofilms and their frequent multidrug-resistant (MDR) phenotypes^5^. The current findings support previous evidence linking biofilm formation to increased antibiotic tolerance in *Vibrio* species. The biofilm matrix acts as a formidable physical and biochemical barrier, limiting the penetration of conventional antimicrobials. In the present study, qualitative CRA screening suggested broader phenotypic variability among *V. fluvialis* isolates, while quantitative MTPA provided the definitive biofilm classification. These data are consistent with those of previous studies, highlighting the elevated biofilm-associated virulence of *V. fluvialis*^59,60^. Such biofilm-mediated tolerance further complicates the treatment of *Vibrio* infections, demanding innovative nonantibiotic approaches. Seasonal variation was found to significantly influence the prevalence of biofilm-forming isolates, with the highest rates observed during the summer (*p* = 0.0075). This seasonal effect is consistent with the literature, suggesting that elevated temperatures promote the expression of biofilm-associated genes, enhance bacterial adhesion, and support EPS synthesis^61,62^. Our data showed that *V*. *fluvialis* biofilm formation peaked at 69.2% during summer, while *V. alginolyticus* reached 30.6%, supporting the hypothesis that environmental stressors, such as heat, play a critical role in virulence modulation. In the same context, recent findings in tropical marine systems emphasize that factors, such as dissolved organic carbon and nutrient load, significantly influence *Vibrio* abundance and community dynamics^63^. Additionally, integrated aquaculture systems have shown seasonal amplification of opportunistic pathogens such as *Vibrio*, underscoring the importance of temporal surveillance strategies^64^. Finally, springing from complementary antimicrobial approaches, disrupting biofilm formation through non-traditional methods shows promise for overcoming the resistance associated with biofilms^65^. However, polymicrobial biofilms are indeed more representative of natural aquaculture conditions^66^; our monospecies biofilm is considered a baseline model that allows characterization of the intrinsic biofilm-forming ability of a specific pathogen without the confounding effects of interspecies interactions^67^.

The current study demonstrated significant formulation-dependent differences in both inhibitory and bactericidal activities against *Vibrio* isolates. These findings reinforce the growing consensus regarding the potential of nanomaterials to enhance disinfectant potency, particularly against biofilm-forming marine pathogens. Our finding of greater resistance in *V. fluvialis,* as shown by the significant association with higher MIC values (χ^2^ = 20.569, *p* = 0.002), conforms to previous studies. Similarly, Mitsuwan, et al.^68^ reported that *V. fluvialis* exhibits the highest multidrug resistance and biofilm-forming capacity among *Vibrio* spp. Species-specific differences in virulence and biofilm production have been demonstrated by Bakhshi, et al.^69^, who reported enhanced biofilm formation and virulence traits in *V. fluvialis* compared with other marine *Vibrios*. Su, et al.^70^ revealed notable antimicrobial resistance in *Vibrio* isolates from aquaculture environments, highlighting the importance of tailored disinfection strategies.

The superior efficacy of TH₄-loaded CuNPs may arise from the combined action of copper ions and quaternary ammonium groups, which potentiate both antibacterial and biofilm-eradicating activities^71^. Therefore, copper–quaternary ammonium nanocomposites are promising candidates for disinfection protocols in aquaculture hatcheries and offshore net cages. The mechanistic basis for the efficacy of copper nanoparticles includes ion release and ROS-mediated microbial damage, as detailed in reviews on copper-based nanomaterials^72^.

AgNPs mixed with hydrogen peroxide also display enhanced antimicrobial potency against multidrug-resistant strains because of oxidative synergy, which increases silver ion release and boosts the generation of reactive oxygen species generation^73–75^. Despite the encouraging results obtained for AgNPs-H₂O₂, environmental safety concerns still remain. The long-term ecological impacts of AgNPs in aquatic systems, including their potential toxicity to non-target organisms and bioaccumulation, warrant thorough investigation before their field application^76,77^. Additionally, sublethal concentrations may exert selective pressure, promote resistance, or affect the microbial community balance^78^. Notably, most MBC/MIC ratios in this study were ≤ 4, indicating bactericidal action, which is consistent with the standard interpretative benchmarks^79^.

Regarding the time-kill assay results, the present investigation demonstrated clear differences in the early bactericidal kinetics of the tested nanoparticle formulations against *V. alginolyticus* and *V. fluvialis*. Both H₂O₂-loaded AgNPs and TH_4_-loaded CuNPs exhibited rapid antimicrobial effects, as reflected by the markedly reduced growth within 15–30 min and complete inhibition by 120 min in most isolates. In contrast, Virkon S-loaded CuNPs failed to achieve comparable reductions, with viable bacterial counts persisting throughout the 120-min interval. These findings are consistent with the concept that nanoparticle biocide hybrids can exert accelerated antimicrobial action through combined mechanisms, including reactive oxygen species (ROS) generation, membrane disruption, and enhanced ion release^75,80^.

The pronounced efficacy of H₂O₂-AgNPs is consistent with that of Martin, et al.^81^, who reported that silver-hydrogen peroxide complexes achieved near-complete bacterial inactivation within 2 h of exposure. This is consistent with previous observations that the H₂O₂-AgNP formulation induced a rapid, time-dependent reduction in bacterial viability, achieving total eradication of multidrug-resistant isolates after 12–24 h of exposure^53^.

Similarly, the rapid decline observed with TH_4_-CuNPs parallels recent evidence that copper nanoparticles functionalized with antimicrobial agents can produce early killing effects against gram-negative pathogens within 30–120 min^82,83^.

Despite the known oxidizing potential of peroxymonosulfate-based disinfectants, the limited effect of Virkon S-loaded CuNPs may be attributed to slower release dynamics or less effective interactions with bacterial membranes under the tested conditions. This is in contrast to the robust early activity of Ag- and Cu-based nanohybrids, supporting the notion that physicochemical characteristics, including particle size, surface charge, and release profile, critically influence the antimicrobial kinetics^84^.

The antimicrobial resistance (AMR) genes found within the *Vibrio* isolates of the seabream confirm the escalating concerns of the emergence and spread of resistance genes within aquaculture ecosystems. The incidence of resistance genes within the *V. alginolyticus,* evidenced herein, was relatively higher, particularly the *sul*, *cat*, and *mphA* genes, than that of *V. fluvialis*, which further confirms that the former has a broader multidrug-resistant (MDR) profile.

The *sul* gene encoding resistance to sulfonamides was found in 80% of *V. alginolyticus* and 40% of *V. fluvialis*. Resistance of *Vibrio* spp. to sulfur-containing antibiotics is a widespread phenomenon that is attributable to the extensive utilization of these antimicrobial agents^85^. In South Africa and Norway, the detection of *sul* genes is common among *Vibrio* species within these two localities and often co-resides with β-lactamase and chloramphenicol acetyltransferase genes^86^. The *cat* gene, linked to resistance to chloramphenicol, was ubiquitous among *V. alginolyticus* isolates and prevalent in *V. fluvialis*. Similar detection of *cat* genes has also been reported in *V. parahaemolyticus* strains isolated from shrimp samples in mainland China, with a high frequency of both *cat* and *sulII* genes (91.8%)^22^. The cat gene is often found widespread even among localities that ban the use of chloramphenicol^87^. The presence of the resistance gene *mphA* in *V. alginolyticus* (40%) and the absence of the *mphA* gene in *V. fluvialis* could indicate a species-dependent acquisition of the gene. The *mphA* resistance gene has also become increasingly common among aquatic bacteria, including marine isolates from Italian aquaculture farms, where the gene was associated with mobile genetic elements involved in spreading resistance genes^88^. Lack of the resistance gene *mphA* in the isolate of the *V. fluvialis* species could be attributed to reduced selective pressures and horizontal gene transfer abilities of the two species.

Intriguingly, no *tet*, *OXA, CTX, or TEM* genes were detected, suggesting that the prevalence of resistance to tetracycline and beta-lactam antibiotics is low within the environment. Though the resistance to antibiotics is well-documented among aquaculture-associated *Vibrio* spp.^24^, The absence of the aforementioned genes within the current isolate may be attributed to the low use of antibiotics within the study site and the changes in the concentration of the antibiotics used over time. Moreover, *V. fluvialis* has been found to harbor the *blaTEM* and *blaOXA* genes within freshwater sites^89^, but such genes were not observed here, indicating geographic and ecological differences in AMR gene distribution.

The coexistence of the *sul*, *cat*, and *mphA* genes within the *V. alginolyticus* genome strengthens the view of its classification as an MDR. This is supported by a previous study that revealed *V. alginolyticus* to be a predominant MDR species within aquaculture ecosystems, which was attributed to the adaptability of the bacteria within the environment and the selective influence of these ecosystems^85^. Moreover, the presence of mobile integrative and conjugative elements within the genome of *V. alginolyticus* that aid in the dispersion of resistance genes within marine bacteria has also been revealed^90^. These also emphasize the ecological involvement of *V. alginolyticus* as a reservoir and vector for the dispersion of AMR genes. Meanwhile, the *V. fluvialis* isolates in this study exhibited fewer AMR genes and lacked macrolide resistance. This is also true for the freshwater and mollusk-associated isolate groups and aligns with the fact that the level of multidrug resistance in *V. fluvialis* is lower than in *V. alginolyticus*^91^. Additionally, it corresponds to the reduced diversity of AMR genes found in the environmental isolates of these species^92^. Such interspecies differences could stem from variation in ecological niches, antibiotic exposure, and horizontal gene transfer potential. Taken together, these findings should be regarded as hypothesis-generating and methodological rather than confirmatory, providing a foundation for future comparative studies incorporating free disinfectants, larger isolate numbers, and in vivo validation.

The practical use of disinfectant-infused nanocomposites in aquaculture systems could likely be achieved through several delivery methods already familiar within standard farm practice and generally align with international guidelines. One obvious option is immersion treatment. In tanks or hatcheries, this may be a practical approach for fish handling, much like the way chemotherapeutants are already applied in routine management^93,94^. In most cases, the process would follow the usual steps used for tank disinfection: the stock is removed, the system is cleaned thoroughly, and then the tanks, pipelines, and associated equipment are filled with the disinfectant solution for the required contact period before draining and rinsing^95,96^. Another possible route is to incorporate these nanocomposites into coatings used on nets and farm equipment. That idea seems especially relevant because it may help create surfaces with inherent antibiofilm activity, which could reduce pathogen attachment and colonization^97^. At the same time, this is not just a matter of convenience. Nets, ropes, and harvest tools are well known to be difficult to sanitize once contamination is established, which is why existing guidance already emphasizes assigning such equipment to specific sites and disinfecting it carefully^98^. From that perspective, surface coating may offer a more preventive strategy. Rather than waiting for contamination to occur and then trying to control it, it aims to reduce the opportunity for transmission in the first place. Water treatment in recirculating aquaculture systems (RAS) also appears to be a feasible application, whether used as a preventive measure or during disease episodes^96^. Even so, this kind of use would probably need tighter control than it may first seem. Suspended solids, for example, can interfere with disinfectant activity, so pre-filtration would still be necessary. After treatment, a neutralization step would also be required, whether through chemical inactivation or activated charcoal filtration, to reduce toxic residues before the water is returned to culture tanks or discharged into the environment^94,99^. So, while the concept looks promising, its success would likely depend less on the material alone and more on how carefully it is integrated into existing treatment protocols.

## Study scope and limitations

The objective of this study was to explore and implement an exploratory approach to nanocomposites loaded with disinfectant against biofilm-forming *Vibrio* spp. isolated from seabream. The nanocomposite formulations were not newly synthesized, and their physicochemical properties have been reported previously. The present work’s innovative aspect lies in its application to *Vibrio* isolates from marine fish with specific biofilm phenotypes, rather than in nanomaterial development. Several limitations should be acknowledged. **Antimicrobial and antibiofilm testing were done on representative isolates of *Vibrio* spp., which restricts population-level inference.** Further, the statistical associations identified should be viewed as preliminary; the limited sample size may reduce the robustness of chi-square tests. **This study did not include any in-vivo or farm-scale validation, and comparing it to parent disinfectants or plain nanoparticles was not within its scope.** The ecotoxicological effects and environmental fate of the nanoparticle formulations have not been evaluated and require careful investigation before practical application.

## Conclusion

Disinfectant-loaded nanoparticles demonstrated potent antimicrobial and antibiofilm properties against biofilm-producing *Vibrio* spp. isolates derived from gilthead seabream, with the highest MIC shown by CuNPs-TH4 and the strongest MBC effect displayed by AgNPs-H_2_O_2_. The performance of the CuNPs-Virkon S was relatively low. **These findings support further investigation of disinfectant-loaded nanoparticle formulations as potential in vitro tools for controlling biofilm-associated *Vibrio* spp., pending in vivo and ecotoxicological validation.** The broader resistome observed in *V. alginolyticus* than in *V. fluvialis* suggests species-specific adaptability and indicates that some *Vibrio* species may accumulate resistance more readily under aquaculture conditions. These species-specific differences in antimicrobial sensitivity and ARG expression highlight the challenges and complexities associated with combating MDR *Vibrio* infections in aquaculture. Seasonal variability showed a peak expression of *Vibrio* spp. during summer, signifying the importance of periodic surveillance. Nanotechnology-disinfection agents, such as copper-quaternary and silver-peroxide nanocomposite formulations, are a promising in vitro approach for biofilm control. Further in vivo studies, environmental safety assessments, and field validation are required before their practical application in aquaculture can be recommended.

## Supplementary Information

Below is the link to the electronic supplementary material.

Supplementary Material 1

Supplementary Material 2

## Data Availability

All data supporting the findings of this study are available within the paper.

## References

[1] Aly, S. M. et al. Studies on Vibrio campbellii as a newly emerging pathogen affecting cultured seabream (Sparus aurata) in Egypt. *Aquacult. Int.* **32**, 1685–1701 (2024).

[2] Teh, L. C. & Sumaila, U. R. Contribution of marine fisheries to worldwide employment. *Fish Fish.* **14**, 77–88 (2013).

[3] Vaseeharan, B. & Thaya, R. Medicinal plant derivatives as immunostimulants: An alternative to chemotherapeutics and antibiotics in aquaculture. *Aquac. Int.* **22**, 1079–1091 (2014).

[4] Abdelaziz, M., Ibrahem, M. D., Ibrahim, M. A., Abu-Elala, N. M. & Abdel-moneam, D. A. Monitoring of different *Vibrio* species affecting marine fishes in Lake Qarun and Gulf of Suez: Phenotypic and molecular characterization. *Egypt. J. Aquat. Res.* **43**, 141–146 (2017).

[5] Arunkumar, M., LewisOscar, F., Thajuddin, N., Pugazhendhi, A. & Nithya, C. In vitro and in vivo biofilm forming *Vibrio* spp: A significant threat in aquaculture. *Process Biochem.* **94**, 213–223 (2020).

[6] Sultan, I. et al. Antibiotics, resistome and resistance mechanisms: A bacterial perspective. *Front. Microbiol.* **9**, 2066 (2018). 30298054 10.3389/fmicb.2018.02066 PMC6160567

[7] Ezzat, M., Mohaeed, G., Ad El-Hak, M. & Wahdan, A. Prevalence and genotypic characterization of *Vibrio alginolyticus*in somemarine fishes. *Suez Canal Vet. Med. J.* **23**, 81–90 (2018).

[8] Liu, L. et al. Investigation of Vibrio alginolyticus, V. harveyi, and V. parahaemolyticus in large yellow croaker, Pseudosciaena crocea (Richardson) reared in Xiangshan Bay China. *Aquac. Rep.* **3**, 220–224 (2016).

[9] Al-Sunaiher, A. E., Ibrahim, A. S. & Al-Salamah, A. A. Association of *Vibrio* species with disease incidence in some cultured fishes in the Kingdom of Saudi Arabia. *World Appl. Sci. J.* **8**, 653–660 (2010).

[10] Ramamurthy, T., Chowdhury, G., Pazhani, G. P. & Shinoda, S. *Vibrio fluvialis*: An emerging human pathogen. *Front. Microbiol.* **5**, 91 (2014). 24653717 10.3389/fmicb.2014.00091 PMC3948065

[11] Vinothkumar, K., Bhardwaj, A. K., Ramamurthy, T. & Niyogi, S. K. Triplex PCR assay for the rapid identification of 3 major *Vibrio* species, *Vibrio cholerae*, *Vibrio parahaemolyticus*, and *Vibrio fluvialis*. *Diagn. Microbiol. Infect. Dis.* **76**, 526–528 (2013). 23706502 10.1016/j.diagmicrobio.2013.04.005

[12] Yiagnisis, M. & Athanassopoulou, F. Bacteria isolated from diseased wild and farmed marine fish in Greece. *Recent Adv. Fish Farms* **27**, 61–69 (2011).

[13] Zaki, V. H., Gala, A. & Eissa, A. Common vibrios affecting thinlip grey mullet (*Liza ramada*). *JEVMA* **78**, 385–402 (2018).

[14] Deng, Y. et al. Prevalence, virulence genes, and antimicrobial resistance of *Vibrio* species isolated from diseased marine fish in South China. *Sci. Rep.* **10**, 14329 (2020). 32868874 10.1038/s41598-020-71288-0 PMC7459350

[15] Schmidt, A. S., Bruun, M. S., Dalsgaard, I., Pedersen, K. & Larsen, J. L. Occurrence of antimicrobial resistance in fish-pathogenic and environmental bacteria associated with four Danish rainbow trout farms. *Appl. Environ. Microbiol.* **66**, 4908–4915 (2000). 11055942 10.1128/aem.66.11.4908-4915.2000 PMC92398

[16] Das, B., Verma, J., Kumar, P., Ghosh, A. & Ramamurthy, T. Antibiotic resistance in *Vibrio cholerae*: Understanding the ecology of resistance genes and mechanisms. *Vaccine* **38**, A83–A92 (2020). 31272870 10.1016/j.vaccine.2019.06.031

[17] Othman, R. *et al.* In *Essentials of aquaculture practices* 139–182 (Springer, 2024).

[18] Ibáñez-Cervantes, G. et al. Ozone and peroxone disinfection of *Toxocara canis* eggs in water. *Trop. Biomed.* **41**(1), 45–51. 10.47665/tb.41.1.006 (2024). 38852133 10.47665/tb.41.1.006

[19] Bögner, D. et al. Hydrogen peroxide oxygenation and disinfection capacity in recirculating aquaculture systems. *Aquacult. Eng.* **92**, 102140 (2021).

[20] Boyce, J. M. Quaternary ammonium disinfectants and antiseptics: Tolerance, resistance and potential impact on antibiotic resistance. *Antimicrob. Resist. Infect. Control.* **12**, 32 (2023). 37055844 10.1186/s13756-023-01241-z PMC10099023

[21] Deng, Y. et al. Global distribution of antimicrobial resistance genes in aquaculture. *One Health Adv.* **3**, 6 (2025).

[22] Zhang, F. et al. Antibiotic resistance and genetic profiles of *Vibrio parahaemolyticus* isolated from farmed Pacific white shrimp (*Litopenaeus vannamei*) in Ningde regions. *Microorganisms* **12**, 152 (2024). 38257979 10.3390/microorganisms12010152 PMC10821069

[23] Costa, W. F., Giambiagi-deMarval, M. & Laport, M. S. Antibiotic and heavy metal susceptibility of non-cholera *Vibrio* isolated from marine sponges and sea urchins: Could they pose a potential risk to public health?. *Antibiotics* **10**, 1561 (2021). 34943773 10.3390/antibiotics10121561 PMC8698511

[24] Sun, Y. et al. Prevalence, antibiotic susceptibility, and genomic analysis of Vibrio alginolyticus isolated from seafood and freshwater products in China. *Front. Microbiol.* **15**, 1381457 (2024). 39050630 10.3389/fmicb.2024.1381457 PMC11266014

[25] Igbinosa, E. O. Detection and antimicrobial resistance of Vibrio isolates in aquaculture environments: Implications for public health. *Microb. Drug Resist.* **22**, 238–245 (2016). 26540391 10.1089/mdr.2015.0169

[26] Chowdhury, G. et al. Emergence of azithromycin resistance mediated by phosphotransferase-encoding mph (A) in diarrheagenic *Vibrio fluvialis*. *mSphere* **4**, 10.1128/msphere. 00215-00219 (2019). 10.1128/mSphere.00215-19 PMC6563354 31189560

[27] Meza-Villezcas, A. et al. Effect of antimicrobial nanocomposites on *Vibrio cholerae* lifestyles: Pellicle biofilm, planktonic and surface-attached biofilm. *PLoS ONE* **14**, e0217869 (2019). 31188854 10.1371/journal.pone.0217869 PMC6561565

[28] Haussler, S. & Fuqua, C. Biofilms 2012: New discoveries and significant wrinkles in a dynamic field. *J. Bacteriol.* **195**, 2947–2958 (2013). 23625847 10.1128/JB.00239-13 PMC3697544

[29] Kim, J. S. et al. Antimicrobial effects of silver nanoparticles. *Nanomedicine (Lond)* **3**, 95–101 (2007). 10.1016/j.nano.2006.12.001 17379174

[30] Morones, J. R. et al. The bactericidal effect of silver nanoparticles. *Nanotechnology* **16**, 2346 (2005). 20818017 10.1088/0957-4484/16/10/059

[31] Schabes-Retchkiman, P. et al. Biosynthesis and characterization of Ti/Ni bimetallic nanoparticles. *Opt. Mater.* **29**, 95–99 (2006).

[32] Davoodbasha, M., Kim, S.-C., Lee, S.-Y. & Kim, J.-W. The facile synthesis of chitosan-based silver nano-biocomposites via a solution plasma process and their potential antimicrobial efficacy. *Arch. Biochem. Biophys.* **605**, 49–58 (2016). 26853839 10.1016/j.abb.2016.01.013

[33] Krishnaraj, C. et al. Synthesis of silver nanoparticles using *Acalypha indica* leaf extracts and its antibacterial activity against water borne pathogens. *Colloids Surf. B Biointerfaces* **76**, 50–56 (2010). 19896347 10.1016/j.colsurfb.2009.10.008

[34] Salem, W. et al. Antibacterial activity of silver and zinc nanoparticles against *Vibrio cholerae* and enterotoxic *Escherichia coli*. *Int. J. Med. Microbiol.* **305**, 85–95 (2015). 25466205 10.1016/j.ijmm.2014.11.005 PMC4300426

[35] Shameli, K., Ahmad, M. B., Al-Mulla, E. A. J., Shabanzadeh, P. & Bagheri, S. Antibacterial effect of silver nanoparticles on talc composites. *Res. Chem. Intermed.* **41**, 251–263 (2015).

[36] Vaseeharan, B., Ramasamy, P. & Chen, J. Antibacterial activity of silver nanoparticles (AgNps) synthesized by tea leaf extracts against pathogenic *Vibrio harveyi* and its protective efficacy on juvenile *Feneropenaeus indicus*. *Lett. Appl. Microbiol.* **50**, 352–356 (2010). 20132435 10.1111/j.1472-765X.2010.02799.x

[37] Agarwala, M., Choudhury, B. & Yadav, R. Comparative study of antibiofilm activity of copper oxide and iron oxide nanoparticles against multidrug resistant biofilm forming uropathogens. *Indian J. Microbiol.* **54**, 365–368 (2014). 24891746 10.1007/s12088-014-0462-z PMC4039716

[38] Cho, K.-H., Park, J.-E., Osaka, T. & Park, S.-G. The study of antimicrobial activity and preservative effects of nanosilver ingredient. *Electrochim. Acta* **51**, 956–960 (2005).

[39] MubarakAli, D. et al. Synthesis and characterization of biocompatibility of tenorite nanoparticles and potential property against biofilm formation. *Saudi Pharm. J.* **23**, 421–428 (2015). 27134545 10.1016/j.jsps.2014.11.007 PMC4834685

[40] Kim, K.-J. et al. Antifungal activity and mode of action of silver nano-particles on Candida albicans. *Biometals* **22**, 235–242 (2009). 18769871 10.1007/s10534-008-9159-2

[41] Melaiye, A. & Youngs, W. J. Vol. 15 125–130 (Taylor & Francis, 2005).

[42] Nadworny, P. L., Wang, J., Tredget, E. E. & Burrell, R. E. Anti-inflammatory activity of nanocrystalline silver in a porcine contact dermatitis model. *Nanomedicine (Lond)* **4**, 241–251. 10.1016/j.nano.2008.04.006 (2008). 10.1016/j.nano.2008.04.006 18550449

[43] Galdiero, S. et al. Silver nanoparticles as potential antiviral agents. *Molecules* **16**, 8894–8918 (2011). 22024958 10.3390/molecules16108894 PMC6264685

[44] Ismail, E. T., El-Son, M. A. M., El-Gohary, F. A. & Zahran, E. Prevalence, genetic diversity, and antimicrobial susceptibility of *Vibrio* spp. infected gilthead sea breams from coastal farms at Damietta, Egypt. *BMC Vet. Res.* **20**, 129. 10.1186/s12917-024-03978-0 (2024). 38561778 10.1186/s12917-024-03978-0 PMC10986055

[45] Elsayed, M. M., Elgohary, F. A., Zakaria, A. I., Elkenany, R. M. & El-Khateeb, A. Y. Novel eradication methods for *Staphylococcus aureus* biofilm in poultry farms and abattoirs using disinfectants loaded onto silver and copper nanoparticles. *Environ. Sci. Pollut. Res. Int.* **27**, 30716–30728. 10.1007/s11356-020-09340-9 (2020). 32468379 10.1007/s11356-020-09340-9

[46] Milanov, D. et al. Slime production and biofilm forming ability by *Staphylococcus aureus* bovine mastitis isolates. *Acta Vet. (Beograd)* **60**, 217–226 (2010).

[47] O’Toole, G. A. Microtiter dish biofilm formation assay. *Journal of visualized experiments: JoVE*, 2437 (2011). 10.3791/2437 PMC3182663 21307833

[48] Merritt, J. H., Kadouri, D. E. & O’Toole, G. A. Growing and analyzing static biofilms. *Curr. Protoc. Microbiol.* **22**, 1B. 1.1-1B. 1.18 (2011). 10.1002/9780471729259.mc01b01s00 PMC4568995 18770545

[49] Stepanović, S. et al. Quantification of biofilm in microtiter plates: Overview of testing conditions and practical recommendations for assessment of biofilm production by staphylococci. *APMIS* **115**, 891–899 (2007). 17696944 10.1111/j.1600-0463.2007.apm_630.x

[50] Wikler, M. A. & Ferraro, M. J. Correction of a reference to clinical laboratory standards institute interpretive criteria. *Clin. Infect. Dis.* **46**, 1798 (2008). 18462124 10.1086/588056

[51] Wu, J. et al. Inhibiting foodborne pathogens *Vibrio parahaemolyticus* and *Listeria monocytogenes* using extracts from traditional medicine: Chinese gallnut, pomegranate peel, Baikal skullcap root and forsythia fruit. *Open Agric.* **3**, 163–170. 10.1515/opag-2018-0017 (2018).

[52] Ayala-Núñez, N. V., Lara Villegas, H. H., del Carmen Ixtepan Turrent, L. & Rodríguez Padilla, C. Silver nanoparticles toxicity and bactericidal effect against methicillin-resistant Staphylococcus aureus: nanoscale does matter. *Nanobiotechnology* **5**, 2-9 (2009).

[53] El-Gohary, F. A. et al. Enhanced antibacterial activity of silver nanoparticles combined with hydrogen peroxide against multidrug-resistant pathogens isolated from dairy farms and beef slaughterhouses in Egypt. *Infect. Drug Resist.*10.2147/IDR.S271261 (2020). 33116668 10.2147/IDR.S271261 PMC7550212

[54] Shehabeldine, A. M. et al. Potential antimicrobial and antibiofilm properties of copper oxide nanoparticles: Time-kill kinetic essay and ultrastructure of pathogenic bacterial cells. *Appl. Biochem. Biotechnol.* **195**, 467–485. 10.1007/s12010-022-04120-2 (2023). 36087233 10.1007/s12010-022-04120-2 PMC9832084

[55] Cavassin, E. D. et al. Comparison of methods to detect the in vitro activity of silver nanoparticles (AgNP) against multidrug resistant bacteria. *J. Nanobiotechnol.* **13**, 64 (2015). 10.1186/s12951-015-0120-6 PMC4593215 26438142

[56] Ahmed, O. & Dablool, A. Quality improvement of the DNA extracted by boiling method in Gram negative bacteria. *Int. J. Bioassays* **6**, 5347–5349. 10.21746/ijbio.2017.04.004 (2017).

[57] Magiorakos, A.-P. et al. Multidrug-resistant, extensively drug-resistant and pandrug-resistant bacteria: an international expert proposal for interim standard definitions for acquired resistance. *Clin. Microbiol. Infect.* **18**, 268–281 (2012). 21793988 10.1111/j.1469-0691.2011.03570.x

[58] Kah Sem, N. A. D., Abd Gani, S., Chong, C. M., Natrah, I. & Shamsi, S. Management and mitigation of vibriosis in aquaculture: nanoparticles as promising alternatives. *Int. J. Mol. Sci.* **24**, 12542 (2023). 37628723 10.3390/ijms241612542 PMC10454253

[59] Liang, P., Cui, X., Du, X., Kan, B. & Liang, W. The virulence phenotypes and molecular epidemiological characteristics of Vibrio fluvialis in China. *Gut pathogens* **5**, 6 (2013). 23522652 10.1186/1757-4749-5-6 PMC3636005

[60] Zheng, J. et al. Identification and characteristics of aptamers against inactivated *Vibrio alginolyticus*. *LWT Food Sci. Technol.* **64**, 1138–1142 (2015).

[61] Beshiru, A. & Igbinosa, E. O. Characterization of extracellular virulence properties and biofilm-formation capacity of *Vibrio* species recovered from ready-to-eat (RTE) shrimps. *Microb. Pathog.* **119**, 93–102 (2018). 29654902 10.1016/j.micpath.2018.04.015

[62] Zhang, B. et al. Spatial and seasonal variations in biofilm formation on microplastics in coastal waters. *Sci. Total Environ.* **770**, 145303 (2021). 33515883 10.1016/j.scitotenv.2021.145303

[63] Wong, Y. Y. et al. Environmental factors that regulate *Vibrio* spp. abundance and community structure in tropical waters. *Anthropocene Coasts* **7**, 21 (2024).

[64] Lu, L. et al. Seasonal dynamics of microbial communities link to summer-autumn aquaculture disease outbreaks in Sanggou Bay. *Front. Mar. Sci.* **12**, 1581190 (2025).

[65] Algburi, A., Comito, N., Kashtanov, D., Dicks, L. M. & Chikindas, M. L. Control of biofilm formation: Antibiotics and beyond. *Appl. Environ. Microbiol.* **83**, e02508-02516 (2017). 27864170 10.1128/AEM.02508-16 PMC5244297

[66] Nithyanand, P., Boya, B. R., Lee, J. H. & Lee, J. Polymicrobial biofilms: Interkingdom interactions, resistance and therapeutic strategies. *Microb. Biotechnol.* **18**, e70218 (2025). 40847578 10.1111/1751-7915.70218 PMC12373980

[67] Chen, P. et al. Characterization of mixed-species biofilm formed by *Vibrio parahaemolyticus* and *Listeria monocytogenes*. *Front. Microbiol.*10.3389/fmicb.2019.02543 (2019). 31787947 10.3389/fmicb.2019.02543 PMC6856058

[68] Mitsuwan, W. et al. Multidrug resistance, biofilm-forming ability, and molecular characterization of *Vibrio* species isolated from foods in Thailand. *Antibiotics* **14**, 235 (2025). 40149046 10.3390/antibiotics14030235 PMC11939528

[69] Bakhshi, B., Afshari, N. & Fallah, F. Enterobacterial repetitive intergenic consensus (ERIC)-PCR analysis as a reliable evidence for suspected *Shigella* spp. outbreaks. *Braz. J. Microbiol.* **49**, 529–533 (2018). 29482996 10.1016/j.bjm.2017.01.014 PMC6066780

[70] Su, J. et al. Prevalence, antibiotic and heavy metal resistance of *Vibrio* spp. isolated from the clam *Meretrix meretrix* at different ages in Geligang, Liaohe estuary in China. *Front. Mar. Sci.* **9**, 1071371 (2022).

[71] Gao, S., Sun, Y., Lu, Z., Jiang, N. & Yao, H. Synergistic antibacterial and biofilm eradication activity of quaternary-ammonium compound with copper ion. *J. Inorg. Biochem.* **243**, 112190 (2023). 36965431 10.1016/j.jinorgbio.2023.112190

[72] Ivanova, I. A., Daskalova, D. S., Yordanova, L. P. & Pavlova, E. L. Copper and copper nanoparticles applications and their role against infections: A minireview. *Processes* **12**, 352 (2024).

[73] Lok, C.-N. et al. Proteomic analysis of the mode of antibacterial action of silver nanoparticles. *J. Proteome Res.* **5**, 916–924 (2006). 16602699 10.1021/pr0504079

[74] Natan, M. & Banin, E. From nano to micro: Using nanotechnology to combat microorganisms and their multidrug resistance. *FEMS Microbiol. Rev.* **41**, 302–322 (2017). 28419240 10.1093/femsre/fux003

[75] Rai, M., Yadav, A. & Gade, A. Silver nanoparticles as a new generation of antimicrobials. *Biotechnol. Adv.* **27**, 76–83 (2009). 18854209 10.1016/j.biotechadv.2008.09.002

[76] Fabrega, J., Luoma, S. N., Tyler, C. R., Galloway, T. S. & Lead, J. R. Silver nanoparticles: Behaviour and effects in the aquatic environment. *Environ. Int.* **37**, 517–531 (2011). 21159383 10.1016/j.envint.2010.10.012

[77] Klaine, S. J. et al. Nanomaterials in the environment: Behavior, fate, bioavailability, and effects. *Environ. Toxicol. Chem.* **27**, 1825–1851 (2008). 19086204 10.1897/08-090.1

[78] Bondarenko, O. et al. Toxicity of Ag, CuO and ZnO nanoparticles to selected environmentally relevant test organisms and mammalian cells in vitro: A critical review. *Arch. Toxicol.* **87**, 1181–1200 (2013). 23728526 10.1007/s00204-013-1079-4 PMC3677982

[79] Ishak, A., Mazonakis, N., Spernovasilis, N., Akinosoglou, K. & Tsioutis, C. Bactericidal versus bacteriostatic antibacterials: Clinical significance, differences and synergistic potential in clinical practice. *J. Antimicrob. Chemother.* **80**, 1–17 (2025). 39471409 10.1093/jac/dkae380 PMC11695898

[80] Pal, S., Tak, Y. K. & Song, J. M. Does the antibacterial activity of silver nanoparticles depend on the shape of the nanoparticle? A study of the gram-negative bacterium *Escherichia coli*. *Appl. Environ. Microbiol.* **73**, 1712–1720 (2007). 17261510 10.1128/AEM.02218-06 PMC1828795

[81] Martin, N. L., Bass, P. & Liss, S. N. Antibacterial properties and mechanism of activity of a novel silver-stabilized hydrogen peroxide. *PLoS ONE* **10**, e0131345. 10.1371/journal.pone.0131345 (2015). 26154263 10.1371/journal.pone.0131345 PMC4496041

[82] Salah, I., Parkin, I. P. & Allan, E. Copper as an antimicrobial agent: Recent advances. *RSC Adv.* **11**, 18179–18186 (2021). 35480904 10.1039/d1ra02149d PMC9033467

[83] Santana, B. M. et al. In vitro bactericidal activity of biogenic copper oxide nanoparticles for *Neisseria gonorrhoeae* with enhanced compatibility for human cells. *ACS Appl. Mater. Interfaces* **16**, 21633–21642. 10.1021/acsami.4c02357 (2024). 38632674 10.1021/acsami.4c02357

[84] Franci, G. et al. Silver nanoparticles as potential antibacterial agents. *Molecules* **20**, 8856–8874 (2015). 25993417 10.3390/molecules20058856 PMC6272636

[85] Fri, J., Ndip, R. N., Njom, H. A. & Clarke, A. M. Antibiotic susceptibility of non-cholera *Vibrios* isolated from farmed and wild marine fish (*Argyrosomus japonicus*), implications for public health. *Microb. Drug Resist.* **24**, 1296–1304 (2018). 29565731 10.1089/mdr.2017.0276

[86] Håkonsholm, F. et al. *Vibrios* from the Norwegian marine environment: Characterization of associated antibiotic resistance and virulence genes. *Microbiolopen* **9**, e1093 (2020). 10.1002/mbo3.1093 PMC7520990 32558371

[87] Gobarah, D. et al. Molecular characterization of antimicrobial resistance of *Vibrio* species isolated from fish in Egypt. *J. Hellen. Vet. Med. Soc.* **74**, 5101–5110 (2023).

[88] Zago, V., Veschetti, L., Patuzzo, C., Malerba, G. & Lleo, M. M. Resistome, mobilome and virulome analysis of *Shewanella algae* and *Vibrio* spp. strains isolated in Italian aquaculture centers. *Microorganisms* **8**, 572 (2020). 32326629 10.3390/microorganisms8040572 PMC7232470

[89] Gxalo, O. et al. Virulence and antibiotic resistance characteristics of *Vibrio* isolates from rustic environmental freshwaters. *Front. Cell. Infect. Microbiol.* **11**, 732001 (2021). 34490150 10.3389/fcimb.2021.732001 PMC8416912

[90] Miguel, B., L, M. L. & O, C. R. Integrating conjugative elements of the SXT/R391 family from fish-isolated Vibrios encode restriction–modification systems that confer resistance to bacteriophages. *FEMS Microbiol. Ecol.* **83**, 457–467 (2013). 22974320 10.1111/1574-6941.12007

[91] Sharma, M. H. et al. Multidrug-resistance of Vibrio species in bivalve mollusks from Southern Thailand: Isolation, identification, pathogenicity, and their sensitivity toward chitooligosaccharide-epigallocatechin-3-gallate conjugate. *Foods* **13**, 2375 (2024). 39123565 10.3390/foods13152375 PMC11311814

[92] Rajpara, N., Nair, M. & Bhardwaj, A. K. A highly promiscuous integron, plasmids, extended spectrum beta lactamases and efflux pumps as factors governing multidrug resistance in a highly drug resistant *Vibrio fluvialis* isolate BD146 from Kolkata, India. *Indian J. Microbiol.* **58**, 60–67 (2018). 29434398 10.1007/s12088-017-0687-8 PMC5801176

[93] Sipos, M., Lipscomb, T., Wood, A., Ramee, S. & DiMaggio, M. Evaluation of three embryo disinfectants on hatching success in four freshwater ornamental fish species. *N. Am. J. Aquac.*10.1002/naaq.10118 (2019).

[94] Liu, D. et al. Towards sustainable water disinfection with peracetic acid in aquaculture: A review. *Rev. Aquac.*10.1111/raq.12915 (2024).

[95] Gupta, S. et al. Recent developments in recirculating aquaculture systems: A review. *Aquac. Res.*10.1155/are/6096671 (2024).

[96] Xiao, R. et al. A review on the research status and development trend of equipment in water treatment processes of recirculating aquaculture systems. *Rev. Aquac.*10.1111/raq.12270 (2018).

[97] Schoina, E., Doulgeraki, A., Miliou, H. & Nychas, G. J. Dynamics of water and biofilm bacterial community composition in a Mediterranean recirculation aquaculture system. *Aquac. J.*10.3390/aquacj2020008 (2022).

[98] Basak, D., Jahan, F., Halim, K., Ali, M. N. & Faruk, M. Biosecurity practices in hatcheries of high value fishes. *Bangladesh J. Fish.*10.52168/bjf.2022.34.2 (2022).

[99] Zhu, Z., Gross, A., Brown, P. B. & Luo, G. Disinfection by-products in aquaculture: Sources, impacts, removal and future research. *Rev. Aquac.* **17**, e70035. 10.1111/raq.70035 (2025).

[100] Chen, S., Zhao, S., White, D.G., Schroeder, C.M., Lu, R., Yang, H., McDermott, P.F., Ayers, S. and Meng, J., .Characterization of multiple-antimicrobial-resistant Salmonella serovars isolated from retail meats. *Applied andEnvironmental Microbiology*, **70**(1), pp.1-7 (2004). 10.1128/AEM.70.1.1-7.2004 PMC321239 14711619

[101] Ahmed, A.M., Furuta, K., Shimomura, K., Kasama, Y. and Shimamoto, T., 2006. Genetic characterization ofmultidrug resistance in Shigella spp. from Japan. *Journal of medical microbiology*, **55**(12), pp.1685-1691 (2006). 10.1099/jmm.0.46725-0 17108272

[102] Ouellette, M., Bissonnette, L. and RoY, P.H., . Precise insertion of antibiotic resistance determinants into Tn21-like transposons: nucleotide sequence of the OXA-1 beta-lactamase gene. *Proceedings of the National Academy ofSciences,* **84**(21), pp.7378-7382 (1987). 10.1073/pnas.84.21.7378 PMC299299 2823258

[103] Nguyen, M.C.P., Woerther, P.L., Bouvet, M., Andremont, A., Leclercq, R. and Canu, A., . Escherichia coli asreservoir for macrolide resistance genes. *Emerging infectious diseases*, **15**(10), p.1648 (2009). 10.3201/eid1510.090696 PMC2866414 19861064

