# Supplementary figures and images for: In vitro comparative evaluation of disinfectant-loaded nanoparticles against biofilm-forming Vibrio spp. isolated from gilthead seabream (Sparus aurata)
Source: Sci Rep. 2026 Apr 15;16:12460. doi: 10.1038/s41598-026-45352-0 (PMC13084003; doi:10.1038/s41598-026-45352-0)

## Slide 1
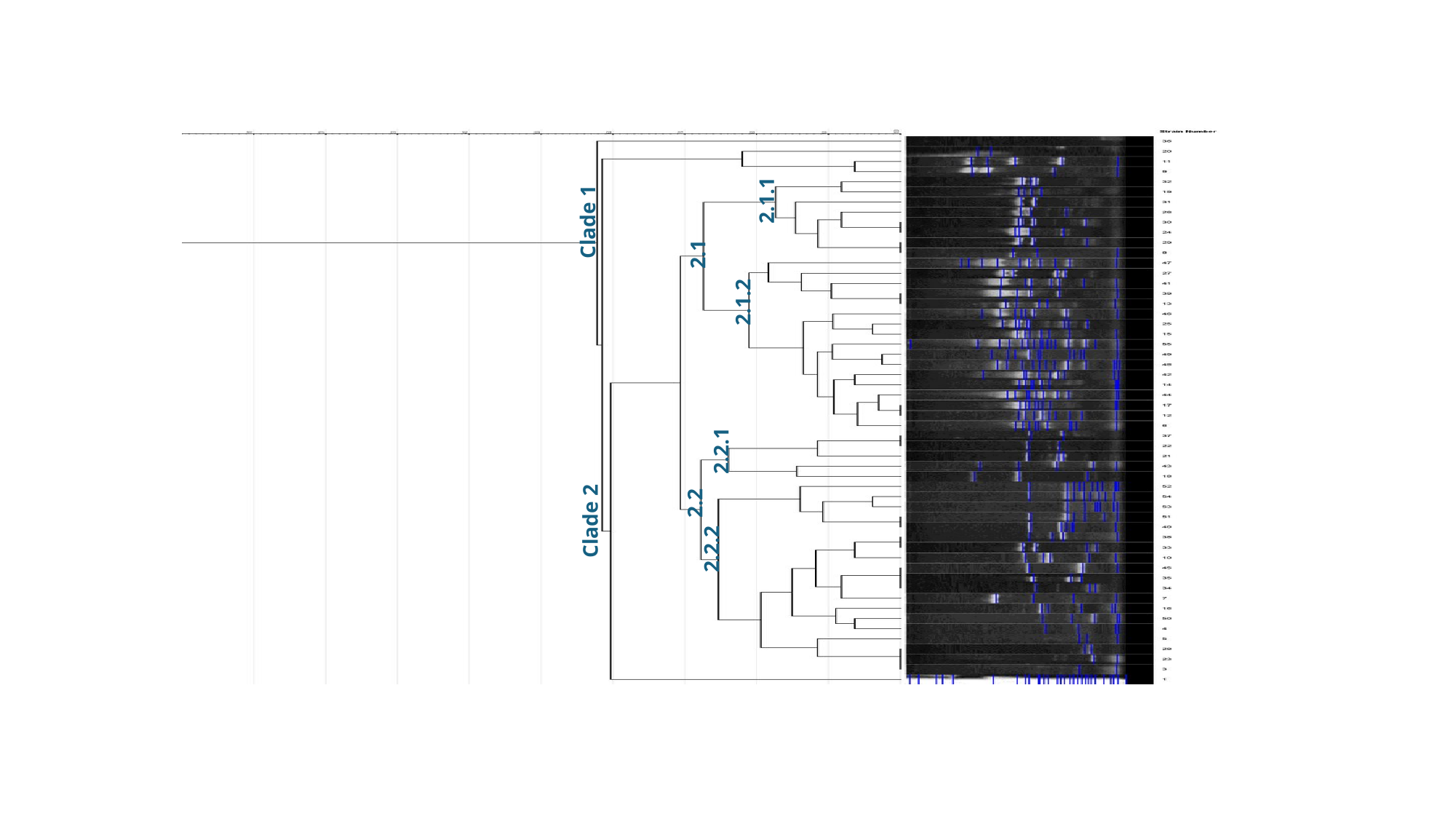

2.1.1
Clade 1
2.1
 2.1.2
2.2.1
 2.2
2.2.2
Clade 2

## Slide 2
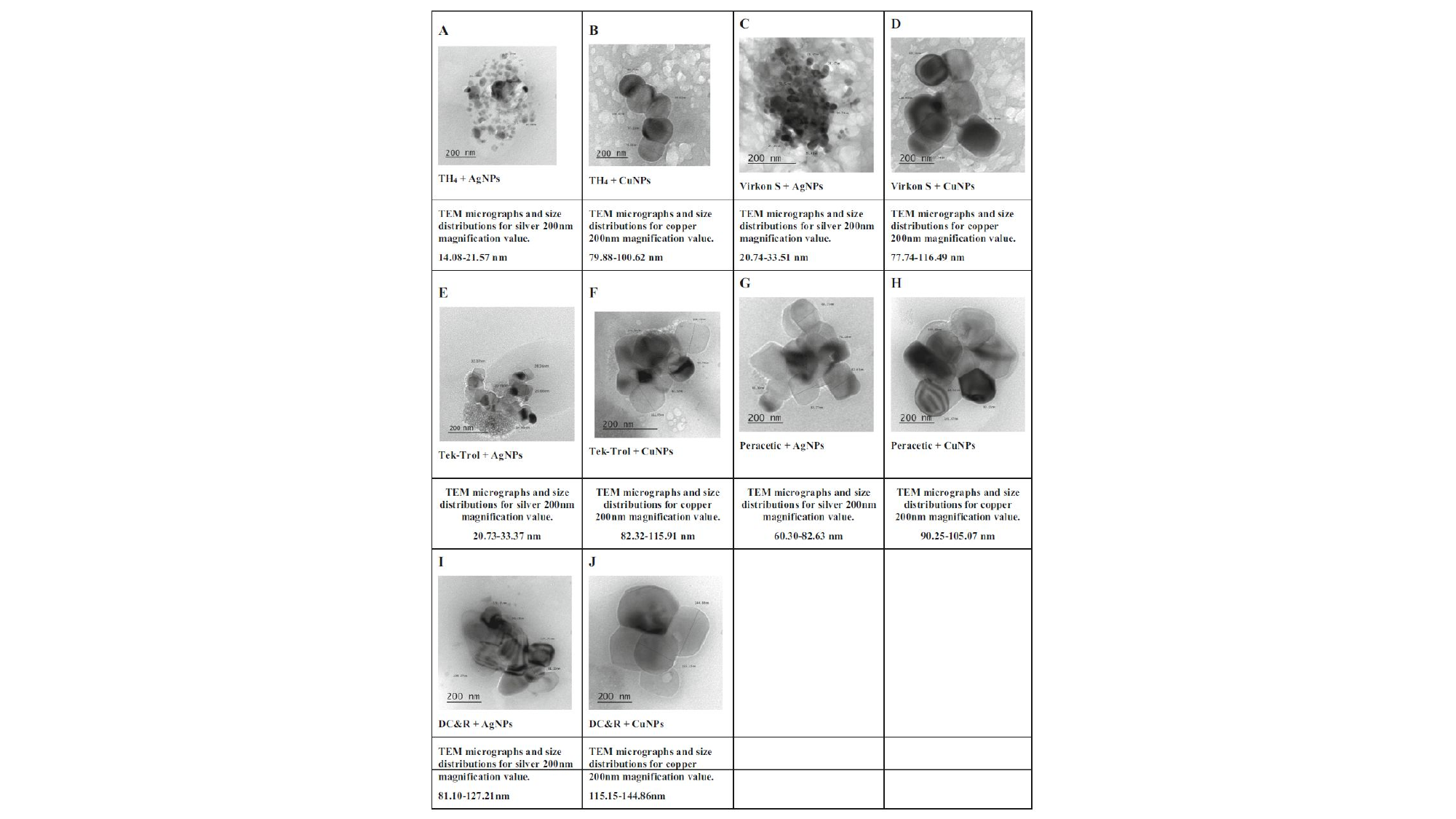

## Slide 3
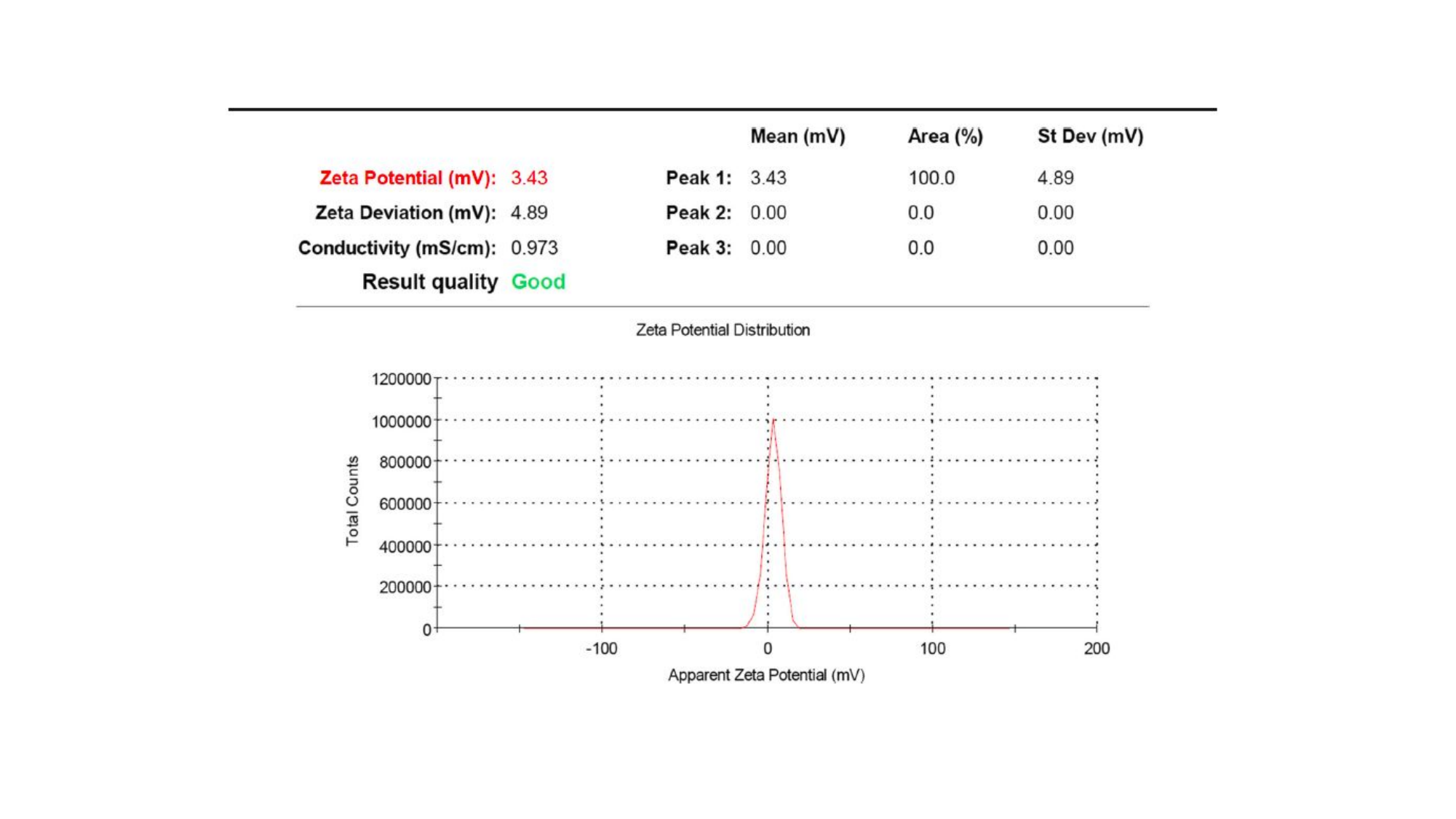

## Slide 4
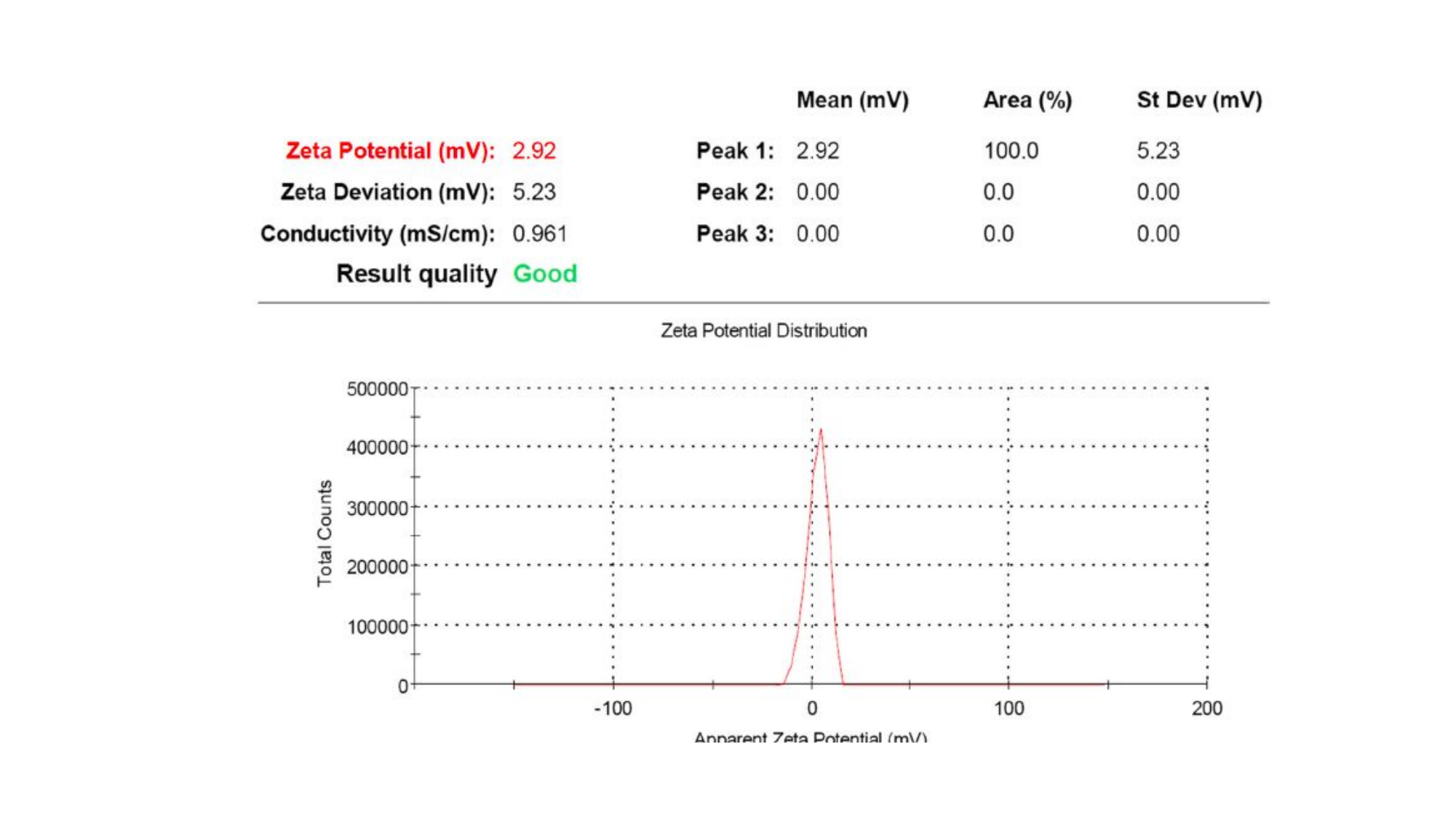

## Slide 5
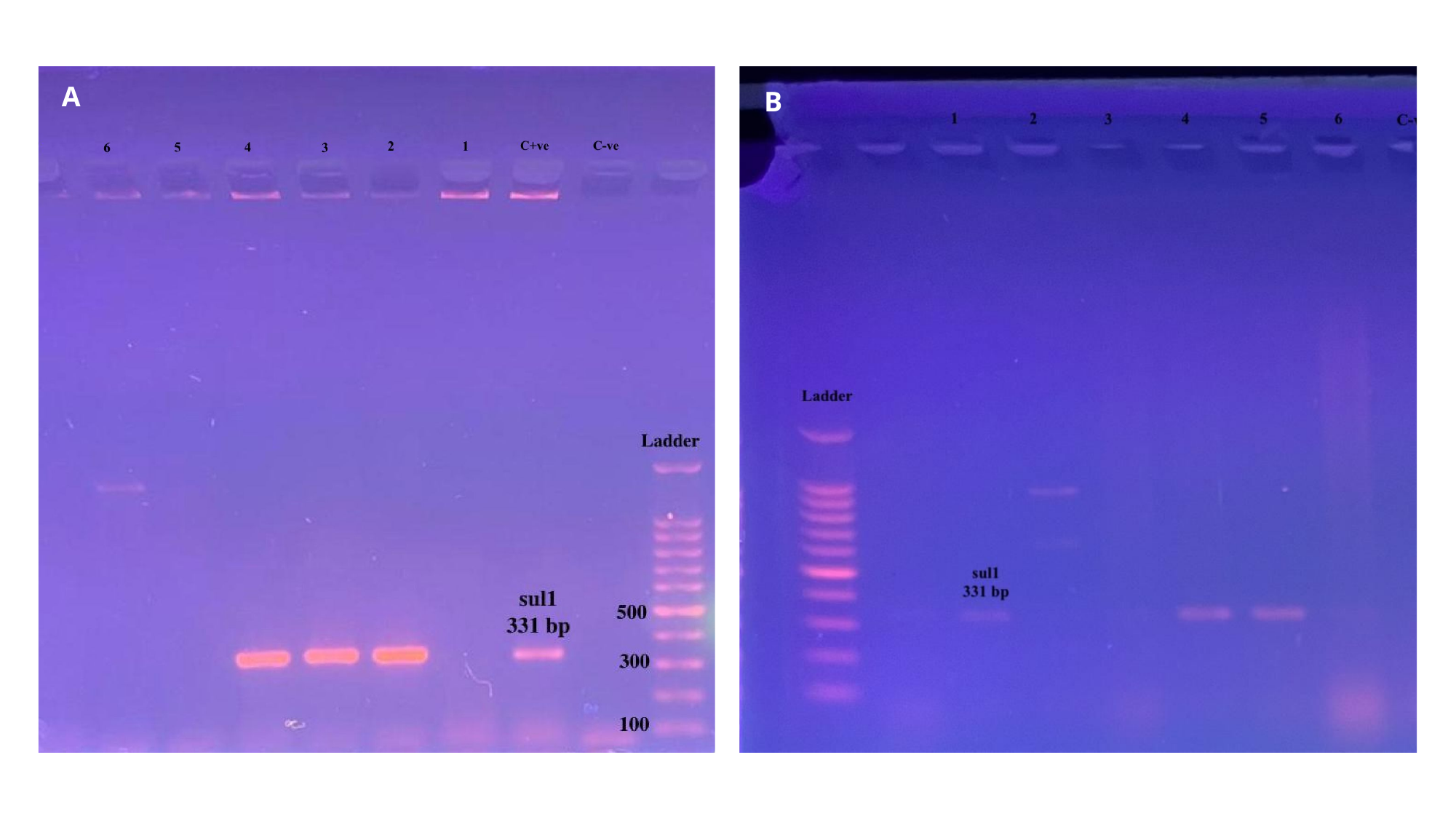

A
B

## Slide 6
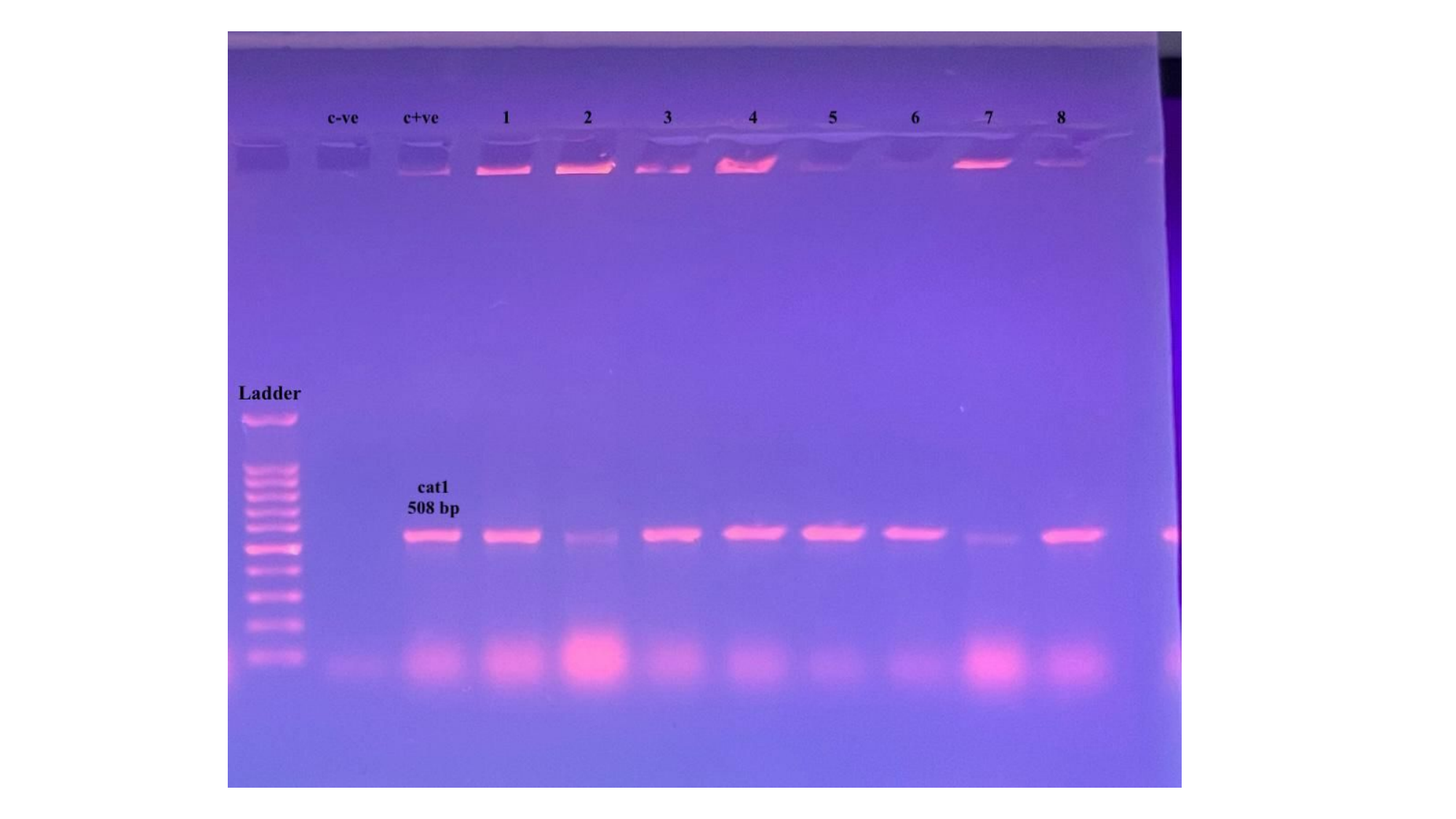

## Slide 7
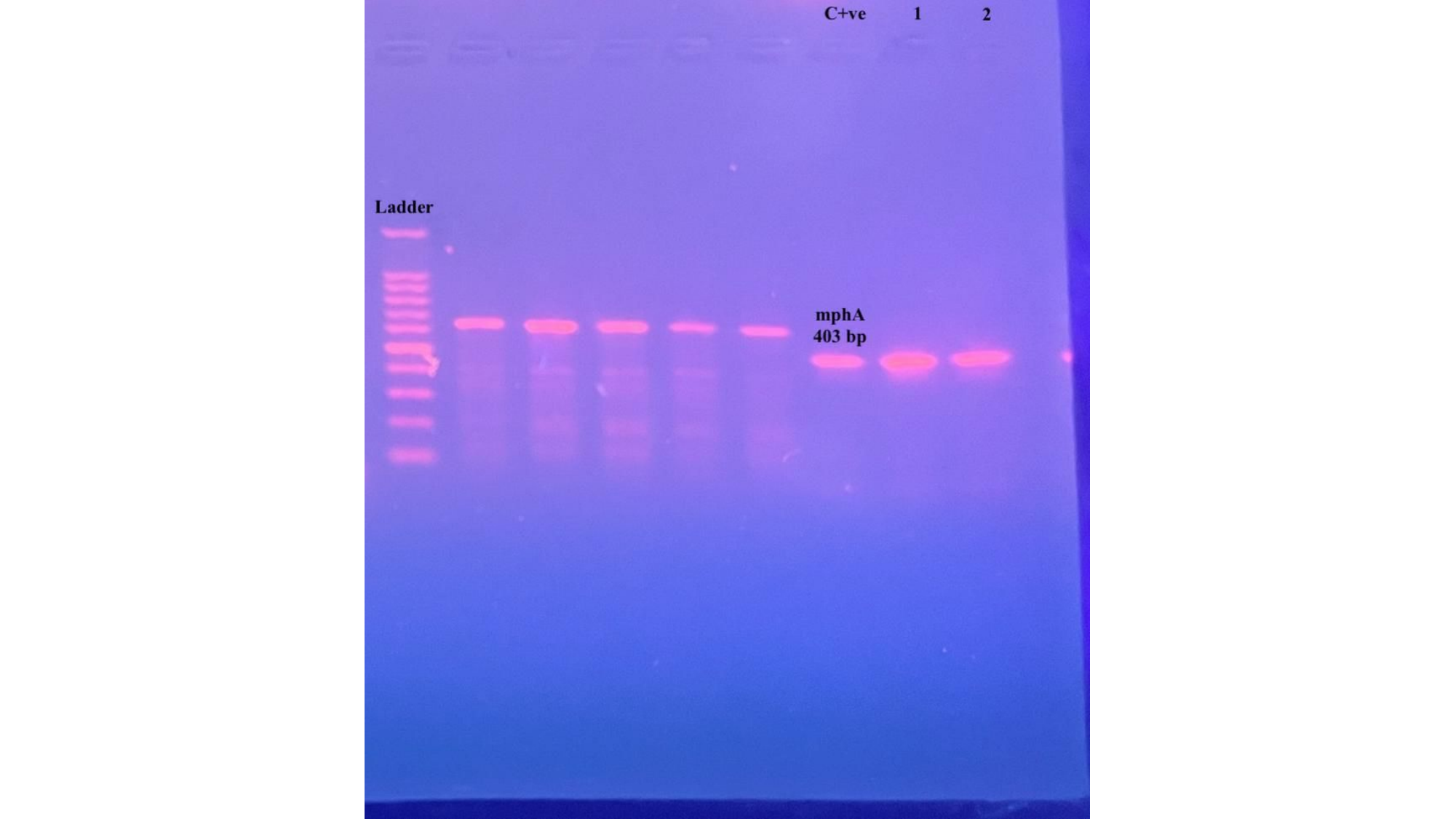

Supplement: Supplementary file 1 — Supplementary Material 1 [file 41598_2026_45352_MOESM1_ESM.pptx]
