# Supplementary material for: In vitro comparative evaluation of disinfectant-loaded nanoparticles against biofilm-forming Vibrio spp. isolated from gilthead seabream (Sparus aurata)
Source: Sci Rep. 2026 Apr 15;16:12460. doi: 10.1038/s41598-026-45352-0 (PMC13084003; doi:10.1038/s41598-026-45352-0)
Supplement: Supplementary file 2 — Supplementary Material 2 [file 41598_2026_45352_MOESM2_ESM.docx]

| Criteria | *V. alginolyticus* | *V. fluvialis* |
| --- | --- | --- |
| Colonies on TCBS | Large dark yellow colony | Yellow colony with shiny margins  *translucent* |
| Gram stain | Gram -ve  straight to slightly curved rod | Gram -ve rods |
| Motility | Motile | Motile |
| Oxidase | + | + |
| Catalase | + | + |
| Anerobic glucose fermentation | + | + |
| Hydrogen sulfide | - | - |
| Ornithine decarboxylase | - | - |
| Arginine bihydrolytic enzyme | - | + |
| Lysine decarboxylase | + | - |
| Urease | - | - |
| Citrate utilization | - | - |
| Nitrate | + | + |
| Indol | + | - |
| Mannitol acid production | + | + |
| Lactose acid production | - | - |
| Mannose acid production | + | + |
| Maltose acid production | + | + |
| Fructose acid production | + | + |
| Xylose acid production | - | - |
| β- galactosidase ONPG | - | - |
| Growth at 6.5% Nacl | + | + |

**Supplementary Table S1.** Biochemical characterization of bacterial isolates retrieved from gilthead seabream (Ismail et 20024).
